# Supplementary material for: The effects of aging and musicianship on the use of auditory streaming cues
Source: PLoS One. 2022 Sep 22;17(9):e0274631. doi: 10.1371/journal.pone.0274631 (PMC9498935; doi:10.1371/journal.pone.0274631)
Supplement: S1 Appendix — (DOCX) [file pone.0274631.s001.docx]

## Supplementary Materials 1 - Model specification details and follow-up t-test results

#### Table 1. Summary of the maximally fitted mixed effects multiple linear regression model predicting d’ when intensity is manipulated including coefficient, standard error, degrees of freedom, t-value and p-value.

|  | Estimate | Std. Error | df | t value | Pr(>\|t\|) |
| --- | --- | --- | --- | --- | --- |
| (Intercept) | 2.3644875 | 0.5994542 | 134.5220 | 3.9444006 | 0.0001282 |
| Level1 | -0.0131490 | 0.5567216 | 1905.6005 | -0.0236186 | 0.9811593 |
| Level2 | -0.1121947 | 0.5567216 | 1905.6005 | -0.2015275 | 0.8403076 |
| Level3 | -0.1384027 | 0.5621718 | 1905.6640 | -0.2461930 | 0.8055594 |
| Level4 | -0.3146534 | 0.5567216 | 1905.6005 | -0.5651899 | 0.5720112 |
| Level5 | -0.6508012 | 0.5568658 | 1905.6025 | -1.1686858 | 0.2426764 |
| Level6 | -0.3704788 | 0.5567216 | 1905.6005 | -0.6654651 | 0.5058336 |
| Level7 | -0.7331093 | 0.5603982 | 1905.6954 | -1.3081935 | 0.1909654 |
| Level8 | -0.2905627 | 0.5604246 | 1905.6956 | -0.5184688 | 0.6041914 |
| Level9 | -0.2670896 | 0.5604246 | 1905.6956 | -0.4765844 | 0.6337128 |
| Level10 | -1.9600293 | 0.5604246 | 1905.6956 | -3.4974008 | 0.0004806 |
| Level11 | -1.8872348 | 0.5616658 | 1905.7139 | -3.3600669 | 0.0007946 |
| Level12 | -2.1070313 | 0.5656153 | 1905.7726 | -3.7252021 | 0.0002008 |
| Level13 | -2.3511152 | 0.5656153 | 1905.7726 | -4.1567390 | 0.0000337 |
| Level14 | -2.1681340 | 0.5656153 | 1905.7726 | -3.8332308 | 0.0001306 |
| Level15 | -2.4816249 | 0.5656153 | 1905.7726 | -4.3874784 | 0.0000121 |
| Level16 | -2.4361137 | 0.5681886 | 1905.8036 | -4.2875092 | 0.0000190 |
| Level17 | -2.5231698 | 0.5689629 | 1905.8081 | -4.4346825 | 0.0000097 |
| Level18 | -2.4463681 | 0.5779382 | 1905.8788 | -4.2329237 | 0.0000242 |
| Level19 | -2.3846514 | 0.5927824 | 1905.9427 | -4.0228110 | 0.0000598 |
| AgeGroup2 | 0.2822658 | 0.9142378 | 138.6336 | 0.3087444 | 0.7579795 |
| GoldMSI-Score | 0.0399440 | 0.0199902 | 140.6299 | 1.9981796 | 0.0476255 |
| WHOPTA | 0.0055851 | 0.0209461 | 47.5773 | 0.2666393 | 0.7908992 |
| Level1:AgeGroup2 | 0.5093264 | 0.8617682 | 1905.6308 | 0.5910248 | 0.5545739 |
| Level2:AgeGroup2 | 0.8795611 | 0.8584638 | 1905.6005 | 1.0245756 | 0.3056935 |
| Level3:AgeGroup2 | 0.6098073 | 0.8620083 | 1905.6275 | 0.7074262 | 0.4793882 |
| Level4:AgeGroup2 | 0.9898741 | 0.8617682 | 1905.6308 | 1.1486547 | 0.2508426 |
| Level5:AgeGroup2 | 0.9606192 | 0.8585573 | 1905.6013 | 1.1188760 | 0.2633341 |
| Level6:AgeGroup2 | 0.9461815 | 0.8584638 | 1905.6005 | 1.1021798 | 0.2705227 |
| Level7:AgeGroup2 | 1.2971738 | 0.8608527 | 1905.6407 | 1.5068476 | 0.1320155 |
| Level8:AgeGroup2 | 0.4342483 | 0.8608698 | 1905.6408 | 0.5044297 | 0.6140178 |
| Level9:AgeGroup2 | 0.2796673 | 0.8608698 | 1905.6408 | 0.3248660 | 0.7453181 |
| Level10:AgeGroup2 | 1.6648725 | 0.8608698 | 1905.6408 | 1.9339422 | 0.0532681 |
| Level11:AgeGroup2 | 2.0525601 | 0.8616784 | 1905.6487 | 2.3820488 | 0.0173142 |
| Level12:AgeGroup2 | 1.3954313 | 0.8671043 | 1905.7069 | 1.6093004 | 0.1077163 |
| Level13:AgeGroup2 | 0.6248594 | 0.8702965 | 1905.8207 | 0.7179846 | 0.4728548 |
| Level14:AgeGroup2 | 0.6041442 | 0.8671043 | 1905.7069 | 0.6967376 | 0.4860520 |
| Level15:AgeGroup2 | 1.0704388 | 0.8683449 | 1905.7186 | 1.2327346 | 0.2178269 |
| Level16:AgeGroup2 | 0.1417497 | 0.8739869 | 1905.7727 | 0.1621874 | 0.8711755 |
| Level17:AgeGroup2 | -0.1451761 | 0.8747385 | 1905.7769 | -0.1659652 | 0.8682020 |
| Level18:AgeGroup2 | -0.3794669 | 0.8806046 | 1905.8086 | -0.4309163 | 0.6665780 |
| Level19:AgeGroup2 | -1.2623848 | 0.8904172 | 1905.8396 | -1.4177454 | 0.1564286 |
| Level1:GoldMSI-Score | -0.0020419 | 0.0188665 | 1905.6005 | -0.1082286 | 0.9138258 |
| Level2:GoldMSI-Score | 0.0061403 | 0.0188665 | 1905.6005 | 0.3254593 | 0.7448692 |
| Level3:GoldMSI-Score | 0.0034885 | 0.0189745 | 1905.6379 | 0.1838538 | 0.8541477 |
| Level4:GoldMSI-Score | 0.0039405 | 0.0188665 | 1905.6005 | 0.2088609 | 0.8345791 |
| Level5:GoldMSI-Score | 0.0129436 | 0.0189221 | 1905.6226 | 0.6840456 | 0.4940296 |
| Level6:GoldMSI-Score | 0.0044757 | 0.0188665 | 1905.6005 | 0.2372325 | 0.8125019 |
| Level7:GoldMSI-Score | 0.0106764 | 0.0189249 | 1905.6452 | 0.5641445 | 0.5727221 |
| Level8:GoldMSI-Score | -0.0017075 | 0.0189405 | 1905.6578 | -0.0901513 | 0.9281765 |
| Level9:GoldMSI-Score | 0.0002967 | 0.0189405 | 1905.6578 | 0.0156642 | 0.9875039 |
| Level10:GoldMSI-Score | 0.0339895 | 0.0189405 | 1905.6578 | 1.7945450 | 0.0728847 |
| Level11:GoldMSI-Score | 0.0338968 | 0.0189454 | 1905.6598 | 1.7891877 | 0.0737434 |
| Level12:GoldMSI-Score | 0.0402639 | 0.0190068 | 1905.6893 | 2.1183940 | 0.0342708 |
| Level13:GoldMSI-Score | 0.0379871 | 0.0190068 | 1905.6893 | 1.9986081 | 0.0457926 |
| Level14:GoldMSI-Score | 0.0275426 | 0.0190068 | 1905.6893 | 1.4490912 | 0.1474767 |
| Level15:GoldMSI-Score | 0.0365509 | 0.0190068 | 1905.6893 | 1.9230429 | 0.0546235 |
| Level16:GoldMSI-Score | 0.0248230 | 0.0190342 | 1905.6994 | 1.3041271 | 0.1923477 |
| Level17:GoldMSI-Score | 0.0223495 | 0.0190343 | 1905.6992 | 1.1741662 | 0.2404752 |
| Level18:GoldMSI-Score | 0.0037769 | 0.0192305 | 1905.7563 | 0.1964036 | 0.8443152 |
| Level19:GoldMSI-Score | -0.0025524 | 0.0195236 | 1905.8088 | -0.1307343 | 0.8959993 |
| AgeGroup2:GoldMSI-Score | -0.0017751 | 0.0322523 | 139.6860 | -0.0550387 | 0.9561863 |
| Level1:AgeGroup2:GoldMSI-Score | -0.0177472 | 0.0304256 | 1905.6158 | -0.5832990 | 0.5597611 |
| Level2:AgeGroup2:GoldMSI-Score | -0.0297763 | 0.0303667 | 1905.6005 | -0.9805568 | 0.3269358 |
| Level3:AgeGroup2:GoldMSI-Score | -0.0198977 | 0.0304339 | 1905.6150 | -0.6538006 | 0.5133191 |
| Level4:AgeGroup2:GoldMSI-Score | -0.0233591 | 0.0304256 | 1905.6158 | -0.7677447 | 0.4427340 |
| Level5:AgeGroup2:GoldMSI-Score | -0.0258466 | 0.0304013 | 1905.6091 | -0.8501820 | 0.3953307 |
| Level6:AgeGroup2:GoldMSI-Score | -0.0192581 | 0.0303667 | 1905.6005 | -0.6341849 | 0.5260363 |
| Level7:AgeGroup2:GoldMSI-Score | -0.0304732 | 0.0304030 | 1905.6178 | -1.0023078 | 0.3163222 |
| Level8:AgeGroup2:GoldMSI-Score | -0.0088004 | 0.0304127 | 1905.6227 | -0.2893664 | 0.7723325 |
| Level9:AgeGroup2:GoldMSI-Score | -0.0046715 | 0.0304127 | 1905.6227 | -0.1536047 | 0.8779377 |
| Level10:AgeGroup2:GoldMSI-Score | -0.0378245 | 0.0304127 | 1905.6227 | -1.2437072 | 0.2137603 |
| Level11:AgeGroup2:GoldMSI-Score | -0.0563127 | 0.0304158 | 1905.6235 | -1.8514316 | 0.0642621 |
| Level12:AgeGroup2:GoldMSI-Score | -0.0406427 | 0.0305005 | 1905.6509 | -1.3325268 | 0.1828465 |
| Level13:AgeGroup2:GoldMSI-Score | -0.0113718 | 0.0305526 | 1905.7047 | -0.3722026 | 0.7097834 |
| Level14:AgeGroup2:GoldMSI-Score | -0.0273937 | 0.0305005 | 1905.6509 | -0.8981402 | 0.3692243 |
| Level15:AgeGroup2:GoldMSI-Score | -0.0534245 | 0.0306487 | 1905.6927 | -1.7431251 | 0.0814730 |
| Level16:AgeGroup2:GoldMSI-Score | -0.0269739 | 0.0307420 | 1905.7185 | -0.8774270 | 0.3803653 |
| Level17:AgeGroup2:GoldMSI-Score | -0.0355122 | 0.0308401 | 1905.7400 | -1.1514919 | 0.2496744 |
| Level18:AgeGroup2:GoldMSI-Score | -0.0118547 | 0.0310103 | 1905.7697 | -0.3822827 | 0.7022943 |
| Level19:AgeGroup2:GoldMSI-Score | 0.0252866 | 0.0311929 | 1905.7904 | 0.8106516 | 0.4176670 |

#### Table 2. Follow-up t-test results comparing pairs of degrees of difficulty when intensity is manipulated including t-statistic, p-value and whether the p-value is statistically significant when Bonferroni correction is applied (* = significant).

| Level1 | Level2 | Statistic | P-value | Significance |
| --- | --- | --- | --- | --- |
| 0 | 1 | 0.14255438424312 | 0.886782033170204 | - |
| 0 | 2 | -0.490072503998802 | 0.624606572108131 | - |
| 0 | 3 | -0.0642784455723457 | 0.948810550624127 | - |
| 0 | 4 | 0.0943020832369484 | 0.924960251889984 | - |
| 0 | 5 | 0.755642645326259 | 0.450714575211905 | - |
| 0 | 6 | 0.150335749841805 | 0.880644028489179 | - |
| 0 | 7 | 0.737082468157858 | 0.461898840643697 | - |
| 0 | 8 | 0.796818741331572 | 0.426466364977516 | - |
| 0 | 9 | 0.556482043758177 | 0.578481461255993 | - |
| 0 | 10 | 2.79749655383818 | 0.00563701645346032 | - |
| 0 | 11 | 2.45129964467686 | 0.0150857608496297 | - |
| 0 | 12 | 2.90152205559746 | 0.00413755551003806 | - |
| 0 | 13 | 3.81236322755358 | 0.000186396158328501 | - |
| 0 | 14 | 5.18185210206799 | 0.000000544233829331481 | * |
| 0 | 15 | 5.89730975111751 | 0.0000000157813976981956 | * |
| 0 | 16 | 7.28763692563865 | 0.0000000000077850354697599 | * |
| 0 | 17 | 9.38984897693125 | 0.0000000000000000148790088902175 | * |
| 0 | 18 | 10.3324837682961 | 0.0000000000000000000318773691204685 | * |
| 0 | 19 | 10.8181159497512 | 0.00000000000000000000127139561015106 | * |
| 1 | 2 | -0.684492538852321 | 0.494422798246936 | - |
| 1 | 3 | -0.220048985835031 | 0.826049147403884 | - |
| 1 | 4 | -0.0486318587348069 | 0.961259456262868 | - |
| 1 | 5 | 0.665227548830563 | 0.506659816004771 | - |
| 1 | 6 | 0.0183643494248257 | 0.985366059726975 | - |
| 1 | 7 | 0.644995994429017 | 0.519654480099039 | - |
| 1 | 8 | 0.707864623104884 | 0.479833117477099 | - |
| 1 | 9 | 0.453933791466501 | 0.650369583035302 | - |
| 1 | 10 | 2.83703113419047 | 0.005028487005826 | - |
| 1 | 11 | 2.46188919555303 | 0.0147145200029851 | - |
| 1 | 12 | 2.93337908785993 | 0.00378184580336597 | - |
| 1 | 13 | 3.88021358527921 | 0.000148019790098467 | - |
| 1 | 14 | 5.3369026671972 | 0.000000278293156950206 | * |
| 1 | 15 | 6.10500417478786 | 0.00000000590566835142107 | * |
| 1 | 16 | 7.56839264971822 | 0.00000000000186615551160774 | * |
| 1 | 17 | 9.87408187790303 | 0.000000000000000000833754623043341 | * |
| 1 | 18 | 10.8814956448432 | 0.0000000000000000000012407658176933 | * |
| 1 | 19 | 11.4222776546712 | 0.0000000000000000000000335961448643846 | * |
| 2 | 3 | 0.449328534043588 | 0.653661832364992 | - |
| 2 | 4 | 0.618372421810329 | 0.537006272917706 | - |
| 2 | 5 | 1.29734151255042 | 0.195987412294611 | - |
| 2 | 6 | 0.650497126078232 | 0.516100276228638 | - |
| 2 | 7 | 1.28303272252672 | 0.200937280464319 | - |
| 2 | 8 | 1.36404323543581 | 0.174047989375137 | - |
| 2 | 9 | 1.072799312621 | 0.284659248256165 | - |
| 2 | 10 | 3.45834546440631 | 0.000666070797575789 | - |
| 2 | 11 | 3.05187268373363 | 0.00260227938651087 | - |
| 2 | 12 | 3.51101755410724 | 0.000562847677393377 | - |
| 2 | 13 | 4.43132078763778 | 0.0000166132895531036 | * |
| 2 | 14 | 5.92364230086659 | 0.0000000154949759480332 | * |
| 2 | 15 | 6.71245010378518 | 0.000000000229739779943389 | * |
| 2 | 16 | 8.17682556641296 | 0.00000000000005119340938206 | * |
| 2 | 17 | 10.555620104311 | 0.00000000000000000000926909134135973 | * |
| 2 | 18 | 11.5702562659687 | 0.0000000000000000000000125394814082102 | * |
| 2 | 19 | 12.1279932550509 | 0.000000000000000000000000293257567301298 | * |
| 3 | 4 | 0.16708766426726 | 0.867463414807437 | - |
| 3 | 5 | 0.856930784268509 | 0.392483020391952 | - |
| 3 | 6 | 0.222023791207139 | 0.824514442551045 | - |
| 3 | 7 | 0.838679000096505 | 0.402622199450709 | - |
| 3 | 8 | 0.905223538443797 | 0.366402971477352 | - |
| 3 | 9 | 0.645755223374711 | 0.519170089699406 | - |
| 3 | 10 | 2.98882670144255 | 0.00315090547588487 | - |
| 3 | 11 | 2.61414046832868 | 0.00965069626855241 | - |
| 3 | 12 | 3.07651455930519 | 0.00240906916082939 | - |
| 3 | 13 | 4.00656467397408 | 0.0000905058137269503 | - |
| 3 | 14 | 5.44745213925725 | 0.000000160182239557832 | * |
| 3 | 15 | 6.20566481258078 | 0.00000000337729495647734 | * |
| 3 | 16 | 7.64864627926198 | 0.00000000000108843928190916 | * |
| 3 | 17 | 9.91333355194109 | 0.000000000000000000578102491962145 | * |
| 3 | 18 | 10.9039354670208 | 0.000000000000000000000915801739382821 | * |
| 3 | 19 | 11.4326689895739 | 0.0000000000000000000000266180170904699 | * |
| 4 | 5 | 0.696108635279094 | 0.487146939797574 | - |
| 4 | 6 | 0.0630852131400891 | 0.949759459200203 | - |
| 4 | 7 | 0.676457916480586 | 0.49950780625481 | - |
| 4 | 8 | 0.738353561851345 | 0.461137883888611 | - |
| 4 | 9 | 0.488789169266361 | 0.625519216310234 | - |
| 4 | 10 | 2.82480734622203 | 0.00520771903189823 | - |
| 4 | 11 | 2.4592979302624 | 0.0147971085670515 | - |
| 4 | 12 | 2.92382542645562 | 0.00388305258903589 | - |
| 4 | 13 | 3.85897378251009 | 0.00015890390889135 | - |
| 4 | 14 | 5.28691142568104 | 0.000000344433064494802 | * |
| 4 | 15 | 6.03752500811312 | 0.00000000808862977052452 | * |
| 4 | 16 | 7.47696086082378 | 0.00000000000293533525873904 | * |
| 4 | 17 | 9.71409315111964 | 0.00000000000000000210327833680486 | * |
| 4 | 18 | 10.6999522457107 | 0.00000000000000000000348774189999136 | * |
| 4 | 19 | 11.2219534475 | 0.000000000000000000000107097617108573 | * |
| 5 | 6 | -0.604906367881373 | 0.54589755653317 | - |
| 5 | 7 | -0.0245961370763887 | 0.980400687237835 | - |
| 5 | 8 | 0.0167206805751603 | 0.986675610905793 | - |
| 5 | 9 | -0.185408610783594 | 0.853090348907984 | - |
| 5 | 10 | 2.05158308078529 | 0.0414746475348812 | - |
| 5 | 11 | 1.73837149549726 | 0.0836708586482486 | - |
| 5 | 12 | 2.20142606393068 | 0.0288683591170856 | - |
| 5 | 13 | 3.14029864032625 | 0.00195882821110926 | - |
| 5 | 14 | 4.47075472918671 | 0.0000131636694941429 | * |
| 5 | 15 | 5.1638752693611 | 0.000000587544687403543 | * |
| 5 | 16 | 6.55230271631661 | 0.000000000496665732936612 | * |
| 5 | 17 | 8.57756166097098 | 0.00000000000000284216559080503 | * |
| 5 | 18 | 9.51216145719906 | 0.00000000000000000753147450610362 | * |
| 5 | 19 | 9.98038187350543 | 0.000000000000000000361630906746399 | * |
| 6 | 7 | 0.585135897444 | 0.559087546938911 | - |
| 6 | 8 | 0.641113797551331 | 0.522155902226628 | - |
| 6 | 9 | 0.408625098449115 | 0.683236342825315 | - |
| 6 | 10 | 2.6477242538563 | 0.00872856889849815 | - |
| 6 | 11 | 2.30842034562921 | 0.0219885594024428 | - |
| 6 | 12 | 2.76106699806476 | 0.00630677587467815 | - |
| 6 | 13 | 3.67727805421066 | 0.00030730893957321 | - |
| 6 | 14 | 5.03820413148878 | 0.00000106302111027552 | * |
| 6 | 15 | 5.7487408834268 | 0.0000000336510714845836 | * |
| 6 | 16 | 7.13810816517815 | 0.0000000000184341308296684 | * |
| 6 | 17 | 9.22320968475587 | 0.0000000000000000441086560916405 | * |
| 6 | 18 | 10.1637188410295 | 0.0000000000000000000983946750199105 | * |
| 6 | 19 | 10.6454124190003 | 0.0000000000000000000040760017856321 | * |
| 7 | 8 | 0.0422545058620103 | 0.966336617643208 | - |
| 7 | 9 | -0.162672805680649 | 0.870935201809269 | - |
| 7 | 10 | 2.0910188942189 | 0.0377548547687365 | - |
| 7 | 11 | 1.77339173677114 | 0.0776795110320577 | - |
| 7 | 12 | 2.23853843397668 | 0.0263128233996757 | - |
| 7 | 13 | 3.18075423195624 | 0.00171895032756241 | - |
| 7 | 14 | 4.52279291442563 | 0.0000105794673086784 | * |
| 7 | 15 | 5.22276692379042 | 0.000000446759765947673 | * |
| 7 | 16 | 6.61990951026598 | 0.000000000346448482925527 | * |
| 7 | 17 | 8.67070085149472 | 0.00000000000000159149737519272 | * |
| 7 | 18 | 9.6130556585341 | 0.00000000000000000395384181793625 | * |
| 7 | 19 | 10.0879518082452 | 0.00000000000000000017972210383999 | * |
| 8 | 9 | -0.207521502392717 | 0.83580951214825 | - |
| 8 | 10 | 2.09633222494905 | 0.0372926619527725 | - |
| 8 | 11 | 1.77025874697946 | 0.0782314288816621 | - |
| 8 | 12 | 2.24365016620541 | 0.0260018120393458 | - |
| 8 | 13 | 3.20009988495395 | 0.00162011457125922 | - |
| 8 | 14 | 4.57133161758013 | 0.00000869746300995075 | * |
| 8 | 15 | 5.28878708692246 | 0.000000332409236476165 | * |
| 8 | 16 | 6.71194896843749 | 0.00000000021828936094498 | * |
| 8 | 17 | 8.82949570157793 | 0.00000000000000061960303780351 | * |
| 8 | 18 | 9.79427081973524 | 0.00000000000000000133033932801495 | * |
| 8 | 19 | 10.2871712793848 | 0.0000000000000000000530577432250213 | * |
| 9 | 10 | 2.20008205418987 | 0.0289137869677487 | - |
| 9 | 11 | 1.88666466676743 | 0.0606354807597327 | - |
| 9 | 12 | 2.34088943175996 | 0.0202314581140967 | - |
| 9 | 13 | 3.26322600201538 | 0.00130456301001755 | - |
| 9 | 14 | 4.57937751716881 | 0.00000823531609255864 | * |
| 9 | 15 | 5.26383127023261 | 0.000000364101465252273 | * |
| 9 | 16 | 6.63282494865391 | 0.000000000314460837607778 | * |
| 9 | 17 | 8.62412979081097 | 0.00000000000000213379950623279 | * |
| 9 | 18 | 9.54353958888848 | 0.00000000000000000616632329323584 | * |
| 9 | 19 | 10.0020938321193 | 0.000000000000000000318807936604513 | * |
| 10 | 11 | -0.224229589711988 | 0.822803401212879 | - |
| 10 | 12 | 0.267499392859529 | 0.789360619060936 | - |
| 10 | 13 | 1.27140541847512 | 0.205109512107214 | - |
| 10 | 14 | 2.48519294950519 | 0.0137705518321071 | - |
| 10 | 15 | 3.11160884474275 | 0.00213267617828545 | - |
| 10 | 16 | 4.48113881857714 | 0.0000125740632185792 | * |
| 10 | 17 | 6.27583743558146 | 0.00000000216845027201983 | * |
| 10 | 18 | 7.17817991065237 | 0.0000000000146526722983961 | * |
| 10 | 19 | 7.59334924631033 | 0.00000000000132592692793439 | * |
| 11 | 12 | 0.47057616908314 | 0.638452276302631 | - |
| 11 | 13 | 1.43506802601269 | 0.152848001031591 | - |
| 11 | 14 | 2.61249065309073 | 0.00966601525706865 | - |
| 11 | 15 | 3.21754947608806 | 0.00150721302057765 | - |
| 11 | 16 | 4.53940832168878 | 0.00000976333027914066 | * |
| 11 | 17 | 6.25450871162612 | 0.00000000247743916021408 | * |
| 11 | 18 | 7.12036325295383 | 0.0000000000209647249814151 | * |
| 11 | 19 | 7.51363419534205 | 0.00000000000221144623030066 | * |
| 12 | 13 | 0.967341899483605 | 0.334555334677056 | - |
| 12 | 14 | 2.1046591690385 | 0.036569950003838 | - |
| 12 | 15 | 2.68692881452658 | 0.00782176198297142 | - |
| 12 | 16 | 3.98858370378172 | 0.0000936541975991661 | - |
| 12 | 17 | 5.62502382060378 | 0.0000000652717098323842 | * |
| 12 | 18 | 6.47161442427356 | 0.000000000815927555645148 | * |
| 12 | 19 | 6.84607188490943 | 0.000000000107471114477728 | * |
| 13 | 14 | 1.05694488366236 | 0.291825701507158 | - |
| 13 | 15 | 1.59393633879323 | 0.112565579236708 | - |
| 13 | 16 | 2.85104690463547 | 0.00482781462297871 | - |
| 13 | 17 | 4.33339573866697 | 0.0000240650157745761 | * |
| 13 | 18 | 5.13954665173075 | 0.000000702717859165692 | * |
| 13 | 19 | 5.47737424602949 | 0.000000143854537769332 | * |
| 14 | 15 | 0.537503222696483 | 0.591521384689078 | - |
| 14 | 16 | 1.85080774344477 | 0.065694561824955 | - |
| 14 | 17 | 3.34037294095228 | 0.00100663110327417 | - |
| 14 | 18 | 4.18121165835654 | 0.0000443938377624879 | * |
| 14 | 19 | 4.52500746972303 | 0.0000107695635653261 | * |
| 15 | 16 | 1.34473200991203 | 0.180266842952517 | - |
| 15 | 17 | 2.83781403356533 | 0.00503284664861247 | - |
| 15 | 18 | 3.69821391034625 | 0.000284637206552955 | - |
| 15 | 19 | 4.04533719740996 | 0.0000765182626085404 | - |
| 16 | 17 | 1.38877222583579 | 0.166544218145253 | - |
| 16 | 18 | 2.24000315434393 | 0.0262762852412855 | - |
| 16 | 19 | 2.5646066216702 | 0.0111319900055849 | - |
| 17 | 18 | 0.916121108640205 | 0.360796228028104 | - |
| 17 | 19 | 1.24926016877026 | 0.213165529821014 | - |
| 18 | 19 | 0.319639066251909 | 0.749612782639839 | - |

#### Table 3. Summary of the maximally fitted mixed effects multiple linear regression model predicting d’ when spectral envelope is manipulated including coefficient, standard error, degrees of freedom, t-value and p-value.

|  | Estimate | Std. Error | df | t value | Pr(>\|t\|) |
| --- | --- | --- | --- | --- | --- |
| (Intercept) | 1.2021151 | 0.6726027 | 123.68405 | 1.7872589 | 0.0763448 |
| Level1 | 0.4672236 | 0.6046347 | 1765.94178 | 0.7727369 | 0.4397815 |
| Level2 | -0.0394990 | 0.6044776 | 1765.93981 | -0.0653440 | 0.9479075 |
| Level3 | 0.0289440 | 0.6050027 | 1765.94625 | 0.0478411 | 0.9618483 |
| Level4 | 0.2638605 | 0.6104033 | 1765.99890 | 0.4322724 | 0.6655962 |
| Level5 | 0.5716035 | 0.6146993 | 1766.08889 | 0.9298912 | 0.3525545 |
| Level6 | -0.2186915 | 0.6166559 | 1766.11997 | -0.3546411 | 0.7229008 |
| Level7 | -0.1510263 | 0.6098262 | 1766.05208 | -0.2476547 | 0.8044304 |
| Level8 | -0.1012794 | 0.6098262 | 1766.05208 | -0.1660792 | 0.8681136 |
| Level9 | -0.6087762 | 0.6103437 | 1766.05871 | -0.9974317 | 0.3186916 |
| Level10 | 0.0362383 | 0.6098612 | 1766.05189 | 0.0594206 | 0.9526239 |
| Level11 | -0.3655920 | 0.6230130 | 1766.19517 | -0.5868128 | 0.5574045 |
| Level12 | -0.3845464 | 0.6389383 | 1766.28106 | -0.6018522 | 0.5473497 |
| Level13 | -0.6297513 | 0.6388338 | 1766.27841 | -0.9857826 | 0.3243748 |
| Level14 | -1.0262190 | 0.6388338 | 1766.27841 | -1.6063944 | 0.1083660 |
| Level15 | -1.2875621 | 0.6426191 | 1766.29490 | -2.0036164 | 0.0452637 |
| Level16 | -2.0940143 | 0.6471304 | 1766.29476 | -3.2358459 | 0.0012353 |
| Level17 | -1.7918637 | 0.6598266 | 1766.28138 | -2.7156583 | 0.0066789 |
| Level18 | -1.5978441 | 0.6598873 | 1766.28390 | -2.4213894 | 0.0155615 |
| Level19 | -2.4213792 | 0.6598266 | 1766.28139 | -3.6697203 | 0.0002500 |
| AgeGroup2 | 0.8317165 | 1.0252558 | 127.21060 | 0.8112283 | 0.4187498 |
| GoldMSI-Score | 0.0534633 | 0.0224481 | 129.72632 | 2.3816425 | 0.0186885 |
| WHOPTA | 0.0128678 | 0.0240410 | 47.82677 | 0.5352425 | 0.5949624 |
| Level1:AgeGroup2 | -0.2195111 | 0.9357726 | 1765.96484 | -0.2345774 | 0.8145640 |
| Level2:AgeGroup2 | 0.0472342 | 0.9320936 | 1765.93961 | 0.0506754 | 0.9595899 |
| Level3:AgeGroup2 | -0.4487225 | 0.9324254 | 1765.94252 | -0.4812422 | 0.6304040 |
| Level4:AgeGroup2 | -1.1108289 | 0.9359474 | 1765.96475 | -1.1868497 | 0.2354465 |
| Level5:AgeGroup2 | -1.6058312 | 0.9418620 | 1766.05088 | -1.7049538 | 0.0883789 |
| Level6:AgeGroup2 | -0.5773265 | 0.9431492 | 1766.06303 | -0.6121264 | 0.5405329 |
| Level7:AgeGroup2 | -0.8341181 | 0.9386931 | 1766.02928 | -0.8885952 | 0.3743417 |
| Level8:AgeGroup2 | -1.6523057 | 0.9489885 | 1766.09710 | -1.7411230 | 0.0818362 |
| Level9:AgeGroup2 | -1.0867853 | 0.9390293 | 1766.03208 | -1.1573496 | 0.2472860 |
| Level10:AgeGroup2 | -2.5274798 | 0.9387158 | 1766.02914 | -2.6924866 | 0.0071592 |
| Level11:AgeGroup2 | -2.1544535 | 0.9639173 | 1766.19561 | -2.2351020 | 0.0255352 |
| Level12:AgeGroup2 | -1.1673757 | 0.9745788 | 1766.23523 | -1.1978260 | 0.2311455 |
| Level13:AgeGroup2 | -0.9785662 | 0.9745103 | 1766.23388 | -1.0041620 | 0.3154381 |
| Level14:AgeGroup2 | -0.6794751 | 0.9870069 | 1766.28084 | -0.6884198 | 0.4912789 |
| Level15:AgeGroup2 | -1.5379631 | 0.9908725 | 1766.28231 | -1.5521301 | 0.1208102 |
| Level16:AgeGroup2 | -0.1910398 | 0.9938042 | 1766.28218 | -0.1922308 | 0.8475835 |
| Level17:AgeGroup2 | 0.1162836 | 1.0168564 | 1766.29209 | 0.1143560 | 0.9089686 |
| Level18:AgeGroup2 | -0.1583215 | 1.0037325 | 1766.28071 | -0.1577327 | 0.8746854 |
| Level19:AgeGroup2 | 0.1288831 | 1.0100164 | 1766.29862 | 0.1276050 | 0.8984761 |
| Level1:GoldMSI-Score | -0.0032641 | 0.0205845 | 1765.97493 | -0.1585728 | 0.8740236 |
| Level2:GoldMSI-Score | 0.0049386 | 0.0205239 | 1765.95180 | 0.2406240 | 0.8098745 |
| Level3:GoldMSI-Score | 0.0019847 | 0.0206230 | 1765.98885 | 0.0962354 | 0.9233445 |
| Level4:GoldMSI-Score | -0.0097546 | 0.0206411 | 1765.98671 | -0.4725808 | 0.6365706 |
| Level5:GoldMSI-Score | -0.0103256 | 0.0207101 | 1766.02832 | -0.4985795 | 0.6181377 |
| Level6:GoldMSI-Score | -0.0000279 | 0.0208142 | 1766.06635 | -0.0013403 | 0.9989307 |
| Level7:GoldMSI-Score | -0.0033403 | 0.0205929 | 1765.99452 | -0.1622049 | 0.8711630 |
| Level8:GoldMSI-Score | -0.0024271 | 0.0205929 | 1765.99452 | -0.1178595 | 0.9061924 |
| Level9:GoldMSI-Score | 0.0089998 | 0.0207092 | 1766.04215 | 0.4345785 | 0.6639215 |
| Level10:GoldMSI-Score | -0.0211316 | 0.0206096 | 1766.00594 | -1.0253312 | 0.3053473 |
| Level11:GoldMSI-Score | -0.0138679 | 0.0208714 | 1766.09837 | -0.6644475 | 0.5064907 |
| Level12:GoldMSI-Score | -0.0212399 | 0.0212486 | 1766.18272 | -0.9995896 | 0.3176461 |
| Level13:GoldMSI-Score | -0.0240398 | 0.0211914 | 1766.16010 | -1.1344105 | 0.2567763 |
| Level14:GoldMSI-Score | -0.0192548 | 0.0211914 | 1766.16010 | -0.9086134 | 0.3636782 |
| Level15:GoldMSI-Score | -0.0197933 | 0.0213287 | 1766.19993 | -0.9280129 | 0.3535277 |
| Level16:GoldMSI-Score | 0.0011938 | 0.0213980 | 1766.20608 | 0.0557913 | 0.9555144 |
| Level17:GoldMSI-Score | -0.0063439 | 0.0215820 | 1766.18313 | -0.2939427 | 0.7688362 |
| Level18:GoldMSI-Score | -0.0137610 | 0.0216378 | 1766.20601 | -0.6359707 | 0.5248779 |
| Level19:GoldMSI-Score | 0.0117430 | 0.0215820 | 1766.18313 | 0.5441104 | 0.5864342 |
| AgeGroup2:GoldMSI-Score | -0.0335880 | 0.0362125 | 128.78071 | -0.9275234 | 0.3553899 |
| Level1:AgeGroup2:GoldMSI-Score | -0.0090204 | 0.0331261 | 1765.98176 | -0.2723042 | 0.7854200 |
| Level2:AgeGroup2:GoldMSI-Score | -0.0085609 | 0.0330523 | 1765.94422 | -0.2590118 | 0.7956563 |
| Level3:AgeGroup2:GoldMSI-Score | 0.0055026 | 0.0330864 | 1765.97613 | 0.1663089 | 0.8679329 |
| Level4:AgeGroup2:GoldMSI-Score | 0.0304839 | 0.0331252 | 1765.95781 | 0.9202615 | 0.3575618 |
| Level5:AgeGroup2:GoldMSI-Score | 0.0341051 | 0.0332388 | 1766.00531 | 1.0260625 | 0.3050027 |
| Level6:AgeGroup2:GoldMSI-Score | 0.0129449 | 0.0333589 | 1766.04734 | 0.3880479 | 0.6980274 |
| Level7:AgeGroup2:GoldMSI-Score | 0.0162583 | 0.0331881 | 1765.99804 | 0.4898828 | 0.6242777 |
| Level8:AgeGroup2:GoldMSI-Score | 0.0310431 | 0.0334607 | 1766.05065 | 0.9277481 | 0.3536650 |
| Level9:AgeGroup2:GoldMSI-Score | 0.0243194 | 0.0332603 | 1766.01674 | 0.7311830 | 0.4647644 |
| Level10:AgeGroup2:GoldMSI-Score | 0.0597751 | 0.0331984 | 1766.00261 | 1.8005417 | 0.0719457 |
| Level11:AgeGroup2:GoldMSI-Score | 0.0433756 | 0.0339059 | 1766.14480 | 1.2792945 | 0.2009614 |
| Level12:AgeGroup2:GoldMSI-Score | 0.0131917 | 0.0342198 | 1766.19347 | 0.3854988 | 0.6999144 |
| Level13:AgeGroup2:GoldMSI-Score | 0.0079016 | 0.0341843 | 1766.18410 | 0.2311477 | 0.8172268 |
| Level14:AgeGroup2:GoldMSI-Score | -0.0092019 | 0.0345205 | 1766.22269 | -0.2665649 | 0.7898353 |
| Level15:AgeGroup2:GoldMSI-Score | 0.0362503 | 0.0346106 | 1766.23619 | 1.0473761 | 0.2950695 |
| Level16:AgeGroup2:GoldMSI-Score | -0.0037584 | 0.0346533 | 1766.23812 | -0.1084582 | 0.9136445 |
| Level17:AgeGroup2:GoldMSI-Score | -0.0149687 | 0.0352253 | 1766.24698 | -0.4249430 | 0.6709300 |
| Level18:AgeGroup2:GoldMSI-Score | 0.0009548 | 0.0348730 | 1766.23926 | 0.0273786 | 0.9781608 |
| Level19:AgeGroup2:GoldMSI-Score | -0.0162316 | 0.0349575 | 1766.24328 | -0.4643237 | 0.6424731 |

#### Table 4. Follow-up t-test results comparing pairs of degrees of difficulty when spectral envelope is manipulated including t-statistic, p-value and whether the p-value is statistically significant when Bonferroni correction is applied (* = significant).

| Level1 | Level2 | Statistic | P-value | Significance |
| --- | --- | --- | --- | --- |
| 0 | 1 | -0.483150633265543 | 0.629503719223471 | - |
| 0 | 2 | -0.0191734560206365 | 0.984721343463401 | - |
| 0 | 3 | 0.393546041694565 | 0.694321711302514 | - |
| 0 | 4 | 0.525802847323137 | 0.599597187650644 | - |
| 0 | 5 | 0.0140551790236822 | 0.988799830386832 | - |
| 0 | 6 | 0.978153349012274 | 0.329208695058399 | - |
| 0 | 7 | 1.34404599640456 | 0.180447521245595 | - |
| 0 | 8 | 1.69169313411996 | 0.0922841988532982 | - |
| 0 | 9 | 1.88700410202639 | 0.0606534367960179 | - |
| 0 | 10 | 3.22196447874016 | 0.00148768878055141 | - |
| 0 | 11 | 3.56146738932874 | 0.000468228529375283 | - |
| 0 | 12 | 4.08751262093761 | 0.0000645770689484431 | - |
| 0 | 13 | 5.39965576901721 | 0.000000194848097558285 | * |
| 0 | 14 | 6.45320081027345 | 0.000000000879044904916156 | * |
| 0 | 15 | 7.16970396105058 | 0.0000000000167717462256628 | * |
| 0 | 16 | 8.03773125280041 | 0.00000000000010488191182166 | * |
| 0 | 17 | 7.3988633909689 | 0.00000000000476217282561892 | * |
| 0 | 18 | 6.97455812787292 | 0.0000000000547593198410443 | * |
| 0 | 19 | 8.2399721384405 | 0.0000000000000330556522447967 | * |
| 1 | 2 | 0.442855102580358 | 0.658333343722879 | - |
| 1 | 3 | 0.856534586006415 | 0.392693345553086 | - |
| 1 | 4 | 0.96956668825683 | 0.333402040002445 | - |
| 1 | 5 | 0.484020286164155 | 0.628893273602232 | - |
| 1 | 6 | 1.41041873556116 | 0.159986843992962 | - |
| 1 | 7 | 1.77765232217498 | 0.0769655399846016 | - |
| 1 | 8 | 2.10219871348864 | 0.036789036000061 | - |
| 1 | 9 | 2.28610928047263 | 0.023310128173658 | - |
| 1 | 10 | 3.59607076779465 | 0.000406747116150003 | - |
| 1 | 11 | 3.91226156403579 | 0.000127144579921741 | - |
| 1 | 12 | 4.43767007184588 | 0.0000153693255088102 | * |
| 1 | 13 | 5.72227178998245 | 0.0000000397106586164192 | * |
| 1 | 14 | 6.74031824836869 | 0.000000000179916649496255 | * |
| 1 | 15 | 7.43579570699064 | 0.00000000000358131697685555 | * |
| 1 | 16 | 8.27391466628084 | 0.0000000000000261052889089857 | * |
| 1 | 17 | 7.65769964474827 | 0.00000000000107951180827725 | * |
| 1 | 18 | 7.24695842313969 | 0.0000000000115401169954368 | * |
| 1 | 19 | 8.46813450660941 | 0.00000000000000864929325780027 | * |
| 2 | 3 | 0.394361282172199 | 0.693719595920456 | - |
| 2 | 4 | 0.521549980232225 | 0.602541467009345 | - |
| 2 | 5 | 0.0321057319906163 | 0.974419132149503 | - |
| 2 | 6 | 0.954437668272696 | 0.341017201467945 | - |
| 2 | 7 | 1.30172111330052 | 0.194487746897046 | - |
| 2 | 8 | 1.63812447077521 | 0.102956556359729 | - |
| 2 | 9 | 1.82756785531825 | 0.0691089235503178 | - |
| 2 | 10 | 3.09891416119427 | 0.00221916251771356 | - |
| 2 | 11 | 3.4381567864464 | 0.000717365199339377 | - |
| 2 | 12 | 3.92594305425182 | 0.000120292273688729 | - |
| 2 | 13 | 5.15023678588501 | 0.00000063937589734787 | * |
| 2 | 14 | 6.16512105509249 | 0.00000000403813078683424 | * |
| 2 | 15 | 6.83141837517615 | 0.00000000011255734727478 | * |
| 2 | 16 | 7.61579631623717 | 0.00000000000135926476638104 | * |
| 2 | 17 | 7.01415665756242 | 0.0000000000440657907597457 | * |
| 2 | 18 | 6.64502032073856 | 0.000000000333089266404781 | * |
| 2 | 19 | 7.79706331403186 | 0.000000000000498412249891121 | * |
| 3 | 4 | 0.144164167903173 | 0.885511863438606 | - |
| 3 | 5 | -0.369095455720811 | 0.71243991430225 | - |
| 3 | 6 | 0.587960801282305 | 0.557229286099259 | - |
| 3 | 7 | 0.934857062802327 | 0.350973852888252 | - |
| 3 | 8 | 1.2919864520564 | 0.197854785869695 | - |
| 3 | 9 | 1.49167595000156 | 0.137384438109117 | - |
| 3 | 10 | 2.78757010477183 | 0.00582006825656183 | - |
| 3 | 11 | 3.14745939467661 | 0.00191307199025826 | - |
| 3 | 12 | 3.63718502499697 | 0.000354806947132561 | - |
| 3 | 13 | 4.88837919695632 | 0.00000212641690842326 | * |
| 3 | 14 | 5.93624711210825 | 0.0000000133442318773424 | * |
| 3 | 15 | 6.62242164177017 | 0.000000000356616076029952 | * |
| 3 | 16 | 7.43463372092292 | 0.00000000000376711322469151 | * |
| 3 | 17 | 6.81195528043894 | 0.000000000132864982670147 | * |
| 3 | 18 | 6.43013346981312 | 0.00000000106954459554366 | * |
| 3 | 19 | 7.62324990572863 | 0.00000000000132413509545968 | * |
| 4 | 5 | -0.499237149620151 | 0.618153669595008 | - |
| 4 | 6 | 0.428793975827262 | 0.668537416391059 | - |
| 4 | 7 | 0.758439606799274 | 0.449073389220341 | - |
| 4 | 8 | 1.11079926789408 | 0.267983164234012 | - |
| 4 | 9 | 1.30787391440718 | 0.192424742669772 | - |
| 4 | 10 | 2.55178087101244 | 0.0114606371500051 | - |
| 4 | 11 | 2.91687960137121 | 0.00395448585246396 | - |
| 4 | 12 | 3.37203015916012 | 0.000902605902386269 | - |
| 4 | 13 | 4.55072947951998 | 0.00000948438221531617 | * |
| 4 | 14 | 5.5715168852793 | 0.000000084633545227631 | * |
| 4 | 15 | 6.21762799818236 | 0.00000000321177616446514 | * |
| 4 | 16 | 6.96274586583364 | 0.0000000000595868869148176 | * |
| 4 | 17 | 6.36824426022464 | 0.00000000154847993239301 | * |
| 4 | 18 | 6.03180985626976 | 0.00000000879903648417804 | * |
| 4 | 19 | 7.13475944007694 | 0.0000000000238124102847616 | * |
| 5 | 6 | 0.940062496038185 | 0.348347959201775 | - |
| 5 | 7 | 1.29421878514186 | 0.197089790705015 | - |
| 5 | 8 | 1.63616089536211 | 0.103402096373864 | - |
| 5 | 9 | 1.82846552472307 | 0.0690092300723215 | - |
| 5 | 10 | 3.12497407053635 | 0.0020457425004252 | - |
| 5 | 11 | 3.46580098980097 | 0.000654815231367722 | - |
| 5 | 12 | 3.96775418406696 | 0.000103160378700719 | - |
| 5 | 13 | 5.22592871499951 | 0.000000454988703942647 | * |
| 5 | 14 | 6.25742134712531 | 0.00000000256059645472158 | * |
| 5 | 15 | 6.94331544338599 | 0.0000000000629992888317243 | * |
| 5 | 16 | 7.75963503174994 | 0.000000000000617419762928949 | * |
| 5 | 17 | 7.14300130461402 | 0.000000000022310627454797 | * |
| 5 | 18 | 6.75329238399042 | 0.000000000192624498573449 | * |
| 5 | 19 | 7.94889641048567 | 0.000000000000216086023636416 | * |
| 6 | 7 | 0.318198534622009 | 0.750676674193988 | - |
| 6 | 8 | 0.685835993629065 | 0.493635315889902 | - |
| 6 | 9 | 0.890305809299934 | 0.37441183531575 | - |
| 6 | 10 | 2.12245148522376 | 0.0350695721349501 | - |
| 6 | 11 | 2.50925402813507 | 0.0129461725492641 | - |
| 6 | 12 | 2.94378083625393 | 0.00365806305112828 | - |
| 6 | 13 | 4.0994137641382 | 0.0000621795700195103 | - |
| 6 | 14 | 5.13200368544484 | 0.000000723965196095845 | * |
| 6 | 15 | 5.76976964771133 | 0.0000000342188972984507 | * |
| 6 | 16 | 6.49593639766249 | 0.00000000085400394903466 | * |
| 6 | 17 | 5.90104241990886 | 0.0000000188494088645366 | * |
| 6 | 18 | 5.58290681663816 | 0.0000000887428255943467 | * |
| 6 | 19 | 6.66365271633653 | 0.000000000362967594551289 | * |
| 7 | 8 | 0.388818460540893 | 0.697832667370085 | - |
| 7 | 9 | 0.603706153215925 | 0.546745764650563 | - |
| 7 | 10 | 1.86209323963031 | 0.0640814755527488 | - |
| 7 | 11 | 2.26963672300579 | 0.0243711936319764 | - |
| 7 | 12 | 2.70706191328922 | 0.007418169675501 | - |
| 7 | 13 | 3.89118171731528 | 0.000138268743422881 | - |
| 7 | 14 | 4.95818119085923 | 0.00000158405589768015 | * |
| 7 | 15 | 5.6167200307918 | 0.0000000709782810675171 | * |
| 7 | 16 | 6.37151861710664 | 0.00000000153657584174343 | * |
| 7 | 17 | 5.75473146144855 | 0.0000000373043063013368 | * |
| 7 | 18 | 5.42370527810921 | 0.000000187256693680926 | * |
| 7 | 19 | 6.54671476696124 | 0.000000000625114976098531 | * |
| 8 | 9 | 0.214657124053958 | 0.830259287235718 | - |
| 8 | 10 | 1.41629565162542 | 0.158278131891206 | - |
| 8 | 11 | 1.83622185414256 | 0.0678921238713165 | - |
| 8 | 12 | 2.23172823312889 | 0.0268204407014501 | - |
| 8 | 13 | 3.33543125603496 | 0.00102898890303434 | - |
| 8 | 14 | 4.37829874568742 | 0.0000199230973029757 | * |
| 8 | 15 | 4.99387293003876 | 0.00000138442984733097 | * |
| 8 | 16 | 5.67591943417368 | 0.0000000566766268404253 | * |
| 8 | 17 | 5.08750126004808 | 0.000000936762223063187 | * |
| 8 | 18 | 4.80712937294009 | 0.00000325550031632845 | * |
| 8 | 19 | 5.83333238002839 | 0.0000000266596885250878 | * |
| 9 | 10 | 1.17406007148204 | 0.241810279779185 | - |
| 9 | 11 | 1.60013158145932 | 0.111235507187617 | - |
| 9 | 12 | 1.97525666696571 | 0.049719401622498 | - |
| 9 | 13 | 3.0405804756928 | 0.00270910026014394 | - |
| 9 | 14 | 4.07174698644314 | 0.0000692534138360512 | - |
| 9 | 15 | 4.66701953893037 | 0.00000597546454780987 | * |
| 9 | 16 | 5.31527437957152 | 0.000000329801049784067 | * |
| 9 | 17 | 4.74022841815751 | 0.00000449207312264128 | * |
| 9 | 18 | 4.48328449033047 | 0.0000132587303208219 | * |
| 9 | 19 | 5.46451119009121 | 0.000000166099623328184 | * |
| 10 | 11 | 0.497156773831675 | 0.61965921083135 | - |
| 10 | 12 | 0.827887717609213 | 0.40879155144312 | - |
| 10 | 13 | 1.86806504853836 | 0.0633217721103173 | - |
| 10 | 14 | 2.96207166698244 | 0.00345297777463443 | - |
| 10 | 15 | 3.55849111051675 | 0.000475605875225209 | - |
| 10 | 16 | 4.18958944553525 | 0.0000441386649949959 | * |
| 10 | 17 | 3.59171198860696 | 0.000426595710867141 | - |
| 10 | 18 | 3.36604535616622 | 0.000934469647990982 | - |
| 10 | 19 | 4.33572965004551 | 0.0000246394336228922 | * |
| 11 | 12 | 0.2880472595 | 0.773640397481896 | - |
| 11 | 13 | 1.23621544654421 | 0.218034155700105 | - |
| 11 | 14 | 2.28529121409609 | 0.0234781735594237 | - |
| 11 | 15 | 2.8298296544101 | 0.0052115377677718 | - |
| 11 | 16 | 3.38001706063688 | 0.000907249733682536 | - |
| 11 | 17 | 2.82156733410707 | 0.00537557324634243 | - |
| 11 | 18 | 2.64714530709624 | 0.00888637231880718 | - |
| 11 | 19 | 3.50647801483394 | 0.000589969992394973 | - |
| 12 | 13 | 0.989767476683711 | 0.323648387715338 | - |
| 12 | 14 | 2.10499186917801 | 0.0367046372111865 | - |
| 12 | 15 | 2.68216537520924 | 0.0080281119605901 | - |
| 12 | 16 | 3.27114179073992 | 0.00130493822980907 | - |
| 12 | 17 | 2.67451114769797 | 0.00824268830493736 | - |
| 12 | 18 | 2.48813197274568 | 0.0138172479364184 | - |
| 12 | 19 | 3.40760527254181 | 0.000827861280510037 | - |
| 13 | 14 | 1.2153559367323 | 0.225835917447414 | - |
| 13 | 15 | 1.81381133613523 | 0.0714209590385682 | - |
| 13 | 16 | 2.41105022636595 | 0.0169605261929081 | - |
| 13 | 17 | 1.76843606726821 | 0.0787779640405224 | - |
| 13 | 18 | 1.60454560107712 | 0.110455266030226 | - |
| 13 | 19 | 2.55094338670632 | 0.0116326730108492 | - |
| 14 | 15 | 0.550903498795497 | 0.58240709471648 | - |
| 14 | 16 | 1.05934747915825 | 0.290954351745313 | - |
| 14 | 17 | 0.438914186935853 | 0.661288696516032 | - |
| 14 | 18 | 0.344911418629786 | 0.730589842124542 | - |
| 14 | 19 | 1.17799762040032 | 0.240488972711072 | - |
| 15 | 16 | 0.492915286456458 | 0.622723509593141 | - |
| 15 | 17 | -0.144740882741653 | 0.88509241907525 | - |
| 15 | 18 | -0.212537379121354 | 0.83195035194621 | - |
| 15 | 19 | 0.608421949924724 | 0.543755934502836 | - |
| 16 | 17 | -0.6776389457821 | 0.498955857461844 | - |
| 16 | 18 | -0.718748841157313 | 0.473343336648747 | - |
| 16 | 19 | 0.116551675710005 | 0.907358828204258 | - |
| 17 | 18 | -0.0792189656755338 | 0.936958149221654 | - |
| 17 | 19 | 0.803608574563817 | 0.422809002927699 | - |
| 18 | 19 | 0.837397843475793 | 0.403644266612298 | - |

#### Table 5. Summary of the maximally fitted mixed effects multiple linear regression model predicting d’ when temporal envelope is manipulated including coefficient, standard error, degrees of freedom, t-value and p-value.

|  | Estimate | Std. Error | df | t value | Pr(>\|t\|) |
| --- | --- | --- | --- | --- | --- |
| (Intercept) | -0.0414266 | 0.5711033 | 132.75924 | -0.0725378 | 0.9422831 |
| Level1 | 0.1105494 | 0.5269496 | 1694.30068 | 0.2097912 | 0.8338559 |
| Level2 | -0.2266330 | 0.5273818 | 1694.29212 | -0.4297323 | 0.6674450 |
| Level3 | -0.0406525 | 0.5273818 | 1694.29212 | -0.0770836 | 0.9385662 |
| Level4 | -0.4417432 | 0.5308576 | 1694.39368 | -0.8321312 | 0.4054522 |
| Level5 | -0.1632295 | 0.5339592 | 1694.48930 | -0.3056966 | 0.7598732 |
| Level6 | 0.2638480 | 0.5337412 | 1694.47836 | 0.4943369 | 0.6211323 |
| Level7 | 0.2683813 | 0.5369059 | 1694.52563 | 0.4998665 | 0.6172340 |
| Level8 | -0.1093156 | 0.5368139 | 1694.52208 | -0.2036377 | 0.8386610 |
| Level9 | -0.6216283 | 0.5372799 | 1694.51975 | -1.1569916 | 0.2474389 |
| Level10 | -0.1843189 | 0.5552386 | 1694.68089 | -0.3319633 | 0.7399580 |
| Level11 | -0.4893975 | 0.5606790 | 1694.68129 | -0.8728657 | 0.3828599 |
| Level12 | -0.6936638 | 0.5686626 | 1694.71082 | -1.2198160 | 0.2227043 |
| Level13 | -0.4440575 | 0.5724971 | 1694.70030 | -0.7756502 | 0.4380638 |
| Level14 | 0.2263260 | 0.5724971 | 1694.70030 | 0.3953313 | 0.6926481 |
| Level15 | -0.0154714 | 0.5725465 | 1694.70312 | -0.0270220 | 0.9784453 |
| Level16 | -0.4058226 | 0.5768007 | 1694.67560 | -0.7035750 | 0.4817941 |
| Level17 | -0.5252956 | 0.5764941 | 1694.67996 | -0.9111900 | 0.3623249 |
| Level18 | -0.3167074 | 0.5768007 | 1694.67560 | -0.5490760 | 0.5830257 |
| Level19 | -0.4456719 | 0.5764941 | 1694.67997 | -0.7730729 | 0.4395870 |
| AgeGroup2 | 0.2021524 | 0.8704835 | 136.49121 | 0.2322300 | 0.8167072 |
| GoldMSI-Score | 0.0562220 | 0.0191070 | 140.52193 | 2.9424754 | 0.0038100 |
| WHOPTA | 0.0126054 | 0.0200950 | 48.34007 | 0.6272885 | 0.5334220 |
| Level1:AgeGroup2 | 0.4246833 | 0.8148648 | 1694.33685 | 0.5211703 | 0.6023163 |
| Level2:AgeGroup2 | 0.2754535 | 0.8124346 | 1694.29230 | 0.3390470 | 0.7346163 |
| Level3:AgeGroup2 | -0.1765594 | 0.8124346 | 1694.29230 | -0.2173214 | 0.8279841 |
| Level4:AgeGroup2 | 0.6745067 | 0.8149145 | 1694.33877 | 0.8277025 | 0.4079556 |
| Level5:AgeGroup2 | 0.5679625 | 0.8198414 | 1694.42144 | 0.6927711 | 0.4885481 |
| Level6:AgeGroup2 | -0.5475822 | 0.8178026 | 1694.39386 | -0.6695775 | 0.5032184 |
| Level7:AgeGroup2 | -0.8059285 | 0.8198720 | 1694.41489 | -0.9829930 | 0.3257512 |
| Level8:AgeGroup2 | -0.2171290 | 0.8198118 | 1694.41339 | -0.2648522 | 0.7911555 |
| Level9:AgeGroup2 | 0.4155229 | 0.8307750 | 1694.53254 | 0.5001630 | 0.6170253 |
| Level10:AgeGroup2 | 0.0830976 | 0.8444519 | 1694.62531 | 0.0984041 | 0.9216230 |
| Level11:AgeGroup2 | 0.9430058 | 0.8480388 | 1694.62721 | 1.1119843 | 0.2663026 |
| Level12:AgeGroup2 | 1.3945399 | 0.8533391 | 1694.63388 | 1.6342155 | 0.1023994 |
| Level13:AgeGroup2 | 1.0590847 | 0.8558993 | 1694.62844 | 1.2373940 | 0.2161123 |
| Level14:AgeGroup2 | -0.5411316 | 0.8560356 | 1694.62960 | -0.6321368 | 0.5273827 |
| Level15:AgeGroup2 | -0.0230527 | 0.8560687 | 1694.63078 | -0.0269285 | 0.9785199 |
| Level16:AgeGroup2 | 0.6302577 | 0.8589200 | 1694.61697 | 0.7337793 | 0.4631848 |
| Level17:AgeGroup2 | 0.7913770 | 0.8587141 | 1694.61894 | 0.9215839 | 0.3568769 |
| Level18:AgeGroup2 | 0.0706958 | 0.8589200 | 1694.61697 | 0.0823078 | 0.9344117 |
| Level19:AgeGroup2 | 0.2900020 | 0.8660722 | 1694.63993 | 0.3348474 | 0.7377817 |
| Level1:GoldMSI-Score | -0.0016641 | 0.0179286 | 1694.33953 | -0.0928173 | 0.9260597 |
| Level2:GoldMSI-Score | 0.0209666 | 0.0180099 | 1694.29212 | 1.1641727 | 0.2445179 |
| Level3:GoldMSI-Score | 0.0036771 | 0.0180099 | 1694.29212 | 0.2041741 | 0.8382419 |
| Level4:GoldMSI-Score | 0.0070332 | 0.0180643 | 1694.33898 | 0.3893426 | 0.6970716 |
| Level5:GoldMSI-Score | 0.0014952 | 0.0181401 | 1694.53216 | 0.0824241 | 0.9343192 |
| Level6:GoldMSI-Score | -0.0189180 | 0.0180461 | 1694.43511 | -1.0483155 | 0.2946428 |
| Level7:GoldMSI-Score | -0.0264072 | 0.0181161 | 1694.47074 | -1.4576661 | 0.1451179 |
| Level8:GoldMSI-Score | -0.0103488 | 0.0180650 | 1694.44023 | -0.5728649 | 0.5668122 |
| Level9:GoldMSI-Score | 0.0076473 | 0.0182003 | 1694.42001 | 0.4201758 | 0.6744103 |
| Level10:GoldMSI-Score | -0.0095601 | 0.0184514 | 1694.57442 | -0.5181248 | 0.6044389 |
| Level11:GoldMSI-Score | -0.0000640 | 0.0186131 | 1694.53146 | -0.0034400 | 0.9972557 |
| Level12:GoldMSI-Score | -0.0010352 | 0.0188177 | 1694.57568 | -0.0550135 | 0.9561342 |
| Level13:GoldMSI-Score | -0.0058298 | 0.0187436 | 1694.59107 | -0.3110322 | 0.7558144 |
| Level14:GoldMSI-Score | -0.0239003 | 0.0187436 | 1694.59107 | -1.2751196 | 0.2024417 |
| Level15:GoldMSI-Score | -0.0132291 | 0.0187897 | 1694.61911 | -0.7040593 | 0.4814926 |
| Level16:GoldMSI-Score | -0.0031011 | 0.0188696 | 1694.53462 | -0.1643435 | 0.8694803 |
| Level17:GoldMSI-Score | -0.0052551 | 0.0187928 | 1694.57921 | -0.2796353 | 0.7797915 |
| Level18:GoldMSI-Score | -0.0161246 | 0.0188696 | 1694.53461 | -0.8545287 | 0.3929329 |
| Level19:GoldMSI-Score | -0.0123717 | 0.0187928 | 1694.57921 | -0.6583243 | 0.5104192 |
| AgeGroup2:GoldMSI-Score | -0.0131253 | 0.0307479 | 138.20190 | -0.4268678 | 0.6701398 |
| Level1:AgeGroup2:GoldMSI-Score | -0.0373740 | 0.0288157 | 1694.32933 | -1.2970021 | 0.1948071 |
| Level2:AgeGroup2:GoldMSI-Score | -0.0383921 | 0.0288483 | 1694.30879 | -1.3308245 | 0.1834258 |
| Level3:AgeGroup2:GoldMSI-Score | -0.0071046 | 0.0288483 | 1694.30879 | -0.2462739 | 0.8055001 |
| Level4:AgeGroup2:GoldMSI-Score | -0.0313299 | 0.0289427 | 1694.35486 | -1.0824798 | 0.2791934 |
| Level5:AgeGroup2:GoldMSI-Score | -0.0296796 | 0.0290950 | 1694.47285 | -1.0200930 | 0.3078300 |
| Level6:AgeGroup2:GoldMSI-Score | 0.0024362 | 0.0290197 | 1694.42210 | 0.0839506 | 0.9331056 |
| Level7:AgeGroup2:GoldMSI-Score | 0.0352876 | 0.0290872 | 1694.44162 | 1.2131670 | 0.2252351 |
| Level8:AgeGroup2:GoldMSI-Score | -0.0101004 | 0.0290554 | 1694.42931 | -0.3476250 | 0.7281650 |
| Level9:AgeGroup2:GoldMSI-Score | -0.0246777 | 0.0293191 | 1694.49342 | -0.8416913 | 0.4000795 |
| Level10:AgeGroup2:GoldMSI-Score | -0.0187803 | 0.0297763 | 1694.60196 | -0.6307143 | 0.5283124 |
| Level11:AgeGroup2:GoldMSI-Score | -0.0405914 | 0.0298768 | 1694.58551 | -1.3586273 | 0.1744455 |
| Level12:AgeGroup2:GoldMSI-Score | -0.0511769 | 0.0300047 | 1694.59932 | -1.7056317 | 0.0882599 |
| Level13:AgeGroup2:GoldMSI-Score | -0.0592835 | 0.0299582 | 1694.60535 | -1.9788727 | 0.0479920 |
| Level14:AgeGroup2:GoldMSI-Score | -0.0013871 | 0.0300278 | 1694.60689 | -0.0461953 | 0.9631600 |
| Level15:AgeGroup2:GoldMSI-Score | -0.0150269 | 0.0300566 | 1694.61784 | -0.4999517 | 0.6171740 |
| Level16:AgeGroup2:GoldMSI-Score | -0.0499435 | 0.0301066 | 1694.58470 | -1.6588868 | 0.0973237 |
| Level17:AgeGroup2:GoldMSI-Score | -0.0408073 | 0.0300586 | 1694.60228 | -1.3575934 | 0.1747735 |
| Level18:AgeGroup2:GoldMSI-Score | -0.0193521 | 0.0301066 | 1694.58470 | -0.6427864 | 0.5204497 |
| Level19:AgeGroup2:GoldMSI-Score | -0.0331526 | 0.0305030 | 1694.61408 | -1.0868634 | 0.2772518 |

#### Table 6. Summary of the maximally fitted mixed effects multiple linear regression model predicting perceptual dissimilarity including coefficient, standard error, degrees of freedom, t-value and p-value.

|  | Estimate | Std. Error | df | t value | Pr(>\|t\|) |
| --- | --- | --- | --- | --- | --- |
| (Intercept) | -0.4301972 | 0.4668614 | 66.91513 | -0.9214665 | 0.3601173 |
| Dissimilarity | 1.3313060 | 0.1411685 | 5680.52838 | 9.4306141 | 0.0000000 |
| FeatureSpec | 0.3144473 | 0.2646894 | 5680.53755 | 1.1879860 | 0.2348886 |
| FeatureTemp | -0.2014620 | 0.2902338 | 5681.09070 | -0.6941369 | 0.4876248 |
| AgeGroup2 | 0.4815614 | 0.7063773 | 66.86283 | 0.6817340 | 0.4977619 |
| GoldMSI-Score | 0.0655402 | 0.0153868 | 66.83533 | 4.2595024 | 0.0000656 |
| WHOPTA | 0.0097273 | 0.0195157 | 47.93436 | 0.4984356 | 0.6204565 |
| Dissimilarity:FeatureSpec | -0.8215267 | 0.1652252 | 5680.25413 | -4.9721625 | 0.0000007 |
| Dissimilarity:FeatureTemp | -0.7449431 | 0.3620700 | 5680.81129 | -2.0574561 | 0.0396879 |
| Dissimilarity:AgeGroup2 | 0.3578174 | 0.2154859 | 5680.44517 | 1.6605148 | 0.0968661 |
| FeatureSpec:AgeGroup2 | -0.8437165 | 0.4035598 | 5680.59361 | -2.0906850 | 0.0366006 |
| FeatureTemp:AgeGroup2 | 0.4493606 | 0.4325046 | 5680.71076 | 1.0389729 | 0.2988615 |
| Dissimilarity:GoldMSI-Score | -0.0095159 | 0.0047045 | 5680.25482 | -2.0227216 | 0.0431486 |
| FeatureSpec:GoldMSI-Score | -0.0296253 | 0.0086737 | 5680.34296 | -3.4155194 | 0.0006411 |
| FeatureTemp:GoldMSI-Score | -0.0187910 | 0.0093977 | 5680.64554 | -1.9995212 | 0.0455995 |
| AgeGroup2:GoldMSI-Score | -0.0254859 | 0.0248518 | 66.65892 | -1.0255150 | 0.3088274 |
| Dissimilarity:FeatureSpec:AgeGroup2 | -0.1940277 | 0.2526763 | 5680.29287 | -0.7678905 | 0.4425842 |
| Dissimilarity:FeatureTemp:AgeGroup2 | -0.9923659 | 0.5429401 | 5680.54221 | -1.8277633 | 0.0676375 |
| Dissimilarity:FeatureSpec:GoldMSI-Score | 0.0144845 | 0.0054966 | 5680.12231 | 2.6351597 | 0.0084326 |
| Dissimilarity:FeatureTemp:GoldMSI-Score | 0.0204285 | 0.0118512 | 5680.49240 | 1.7237513 | 0.0848072 |
| Dissimilarity:AgeGroup2:GoldMSI-Score | 0.0003150 | 0.0075734 | 5680.28534 | 0.0415974 | 0.9668211 |
| FeatureSpec:AgeGroup2:GoldMSI-Score | 0.0144143 | 0.0140373 | 5680.43701 | 1.0268563 | 0.3045318 |
| FeatureTemp:AgeGroup2:GoldMSI-Score | -0.0296438 | 0.0151086 | 5680.56500 | -1.9620468 | 0.0498058 |
| Dissimilarity:FeatureSpec:AgeGroup2:GoldMSI-Score | -0.0032273 | 0.0088620 | 5680.15929 | -0.3641788 | 0.7157381 |
| Dissimilarity:FeatureTemp:AgeGroup2:GoldMSI-Score | 0.0285107 | 0.0190520 | 5680.43824 | 1.4964643 | 0.1345883 |
