# Supplementary material for: The effects of aging and musicianship on the use of auditory streaming cues
Source: PLoS One. 2022 Sep 22;17(9):e0274631. doi: 10.1371/journal.pone.0274631 (PMC9498935; doi:10.1371/journal.pone.0274631)
Supplement: S2 Appendix — (DOCX) [file pone.0274631.s002.docx]

## Supplementary Materials 2 - Summary statstics

#### Table 1. Summary statistics (mean, standard deviation) of d’ by level, age group, musicianship, and feature.

| Level | Age Group | Musicianship | Feature | Mean d’ | SD d’ |
| --- | --- | --- | --- | --- | --- |
| 1 | Older | Musicians | Intensity | 4.04 | 1.39 |
| 1 | Older | Musicians | Spectral env. | 3.01 | 1.88 |
| 1 | Older | Musicians | Temporal env. | 1.75 | 1.77 |
| 1 | Older | Nonmusicians | Intensity | 3.35 | 1.85 |
| 1 | Older | Nonmusicians | Spectral env. | 2.52 | 2.10 |
| 1 | Older | Nonmusicians | Temporal env. | 1.11 | 1.79 |
| 1 | Younger | Musicians | Intensity | 3.98 | 1.61 |
| 1 | Younger | Musicians | Spectral env. | 3.48 | 1.64 |
| 1 | Younger | Musicians | Temporal env. | 2.16 | 2.32 |
| 1 | Younger | Nonmusicians | Intensity | 2.88 | 1.90 |
| 1 | Younger | Nonmusicians | Spectral env. | 1.84 | 1.93 |
| 1 | Younger | Nonmusicians | Temporal env. | 0.74 | 1.70 |
| 2 | Older | Musicians | Intensity | 3.98 | 1.28 |
| 2 | Older | Musicians | Spectral env. | 2.59 | 2.19 |
| 2 | Older | Musicians | Temporal env. | 0.83 | 2.05 |
| 2 | Older | Nonmusicians | Intensity | 3.41 | 1.64 |
| 2 | Older | Nonmusicians | Spectral env. | 2.71 | 1.94 |
| 2 | Older | Nonmusicians | Temporal env. | 1.15 | 1.41 |
| 2 | Younger | Musicians | Intensity | 3.96 | 1.02 |
| 2 | Younger | Musicians | Spectral env. | 3.79 | 1.64 |
| 2 | Younger | Musicians | Temporal env. | 2.38 | 2.21 |
| 2 | Younger | Nonmusicians | Intensity | 2.76 | 1.71 |
| 2 | Younger | Nonmusicians | Spectral env. | 2.22 | 2.30 |
| 2 | Younger | Nonmusicians | Temporal env. | 0.68 | 1.61 |
| 3 | Older | Musicians | Intensity | 4.08 | 1.22 |
| 3 | Older | Musicians | Spectral env. | 2.82 | 2.37 |
| 3 | Older | Musicians | Temporal env. | 1.15 | 1.89 |
| 3 | Older | Nonmusicians | Intensity | 3.66 | 1.43 |
| 3 | Older | Nonmusicians | Spectral env. | 2.54 | 2.02 |
| 3 | Older | Nonmusicians | Temporal env. | 0.94 | 1.69 |
| 3 | Younger | Musicians | Intensity | 4.19 | 0.96 |
| 3 | Younger | Musicians | Spectral env. | 3.67 | 1.61 |
| 3 | Younger | Musicians | Temporal env. | 2.78 | 2.08 |
| 3 | Younger | Nonmusicians | Intensity | 2.76 | 1.82 |
| 3 | Younger | Nonmusicians | Spectral env. | 1.79 | 2.31 |
| 3 | Younger | Nonmusicians | Temporal env. | 0.76 | 1.49 |
| 4 | Older | Musicians | Intensity | 4.05 | 1.22 |
| 4 | Older | Musicians | Spectral env. | 2.55 | 2.33 |
| 4 | Older | Musicians | Temporal env. | 1.48 | 1.85 |
| 4 | Older | Nonmusicians | Intensity | 3.46 | 1.52 |
| 4 | Older | Nonmusicians | Spectral env. | 2.43 | 2.08 |
| 4 | Older | Nonmusicians | Temporal env. | 0.80 | 1.76 |
| 4 | Younger | Musicians | Intensity | 3.97 | 1.32 |
| 4 | Younger | Musicians | Spectral env. | 3.55 | 1.68 |
| 4 | Younger | Musicians | Temporal env. | 2.47 | 2.21 |
| 4 | Younger | Nonmusicians | Intensity | 2.80 | 1.87 |
| 4 | Younger | Nonmusicians | Spectral env. | 1.83 | 1.82 |
| 4 | Younger | Nonmusicians | Temporal env. | 0.54 | 1.22 |
| 5 | Older | Musicians | Intensity | 4.17 | 1.13 |
| 5 | Older | Musicians | Spectral env. | 2.58 | 2.05 |
| 5 | Older | Musicians | Temporal env. | 1.13 | 1.76 |
| 5 | Older | Nonmusicians | Intensity | 3.60 | 1.52 |
| 5 | Older | Nonmusicians | Spectral env. | 2.27 | 2.40 |
| 5 | Older | Nonmusicians | Temporal env. | 0.98 | 1.96 |
| 5 | Younger | Musicians | Intensity | 3.74 | 1.43 |
| 5 | Younger | Musicians | Spectral env. | 3.32 | 2.08 |
| 5 | Younger | Musicians | Temporal env. | 2.03 | 1.95 |
| 5 | Younger | Nonmusicians | Intensity | 2.69 | 1.83 |
| 5 | Younger | Nonmusicians | Spectral env. | 1.97 | 2.09 |
| 5 | Younger | Nonmusicians | Temporal env. | 0.37 | 1.41 |
| 6 | Older | Musicians | Intensity | 4.03 | 1.37 |
| 6 | Older | Musicians | Spectral env. | 2.52 | 2.25 |
| 6 | Older | Musicians | Temporal env. | 1.25 | 1.49 |
| 6 | Older | Nonmusicians | Intensity | 3.33 | 1.83 |
| 6 | Older | Nonmusicians | Spectral env. | 2.22 | 2.16 |
| 6 | Older | Nonmusicians | Temporal env. | 1.04 | 1.56 |
| 6 | Younger | Musicians | Intensity | 3.70 | 1.67 |
| 6 | Younger | Musicians | Spectral env. | 3.54 | 1.66 |
| 6 | Younger | Musicians | Temporal env. | 1.95 | 2.22 |
| 6 | Younger | Nonmusicians | Intensity | 2.49 | 1.83 |
| 6 | Younger | Nonmusicians | Spectral env. | 2.38 | 1.99 |
| 6 | Younger | Nonmusicians | Temporal env. | 0.67 | 1.89 |
| 7 | Older | Musicians | Intensity | 4.09 | 1.19 |
| 7 | Older | Musicians | Spectral env. | 2.27 | 2.02 |
| 7 | Older | Musicians | Temporal env. | 1.06 | 2.09 |
| 7 | Older | Nonmusicians | Intensity | 3.70 | 1.79 |
| 7 | Older | Nonmusicians | Spectral env. | 2.44 | 2.19 |
| 7 | Older | Nonmusicians | Temporal env. | 0.52 | 1.43 |
| 7 | Younger | Musicians | Intensity | 3.76 | 1.41 |
| 7 | Younger | Musicians | Spectral env. | 3.03 | 2.06 |
| 7 | Younger | Musicians | Temporal env. | 1.88 | 2.12 |
| 7 | Younger | Nonmusicians | Intensity | 2.58 | 2.14 |
| 7 | Younger | Nonmusicians | Spectral env. | 1.84 | 2.08 |
| 7 | Younger | Nonmusicians | Temporal env. | 0.70 | 1.59 |
| 8 | Older | Musicians | Intensity | 3.86 | 1.49 |
| 8 | Older | Musicians | Spectral env. | 2.09 | 2.10 |
| 8 | Older | Musicians | Temporal env. | 1.74 | 1.87 |
| 8 | Older | Nonmusicians | Intensity | 3.65 | 1.86 |
| 8 | Older | Nonmusicians | Spectral env. | 2.18 | 1.97 |
| 8 | Older | Nonmusicians | Temporal env. | 0.67 | 1.20 |
| 8 | Younger | Musicians | Intensity | 3.51 | 1.55 |
| 8 | Younger | Musicians | Spectral env. | 3.06 | 1.97 |
| 8 | Younger | Musicians | Temporal env. | 1.47 | 2.10 |
| 8 | Younger | Nonmusicians | Intensity | 2.52 | 1.79 |
| 8 | Younger | Nonmusicians | Spectral env. | 1.84 | 1.96 |
| 8 | Younger | Nonmusicians | Temporal env. | 0.72 | 1.79 |
| 9 | Older | Musicians | Intensity | 3.73 | 1.57 |
| 9 | Older | Musicians | Spectral env. | 2.00 | 2.10 |
| 9 | Older | Musicians | Temporal env. | 0.89 | 1.88 |
| 9 | Older | Nonmusicians | Intensity | 3.41 | 1.61 |
| 9 | Older | Nonmusicians | Spectral env. | 1.62 | 2.07 |
| 9 | Older | Nonmusicians | Temporal env. | 0.44 | 1.32 |
| 9 | Younger | Musicians | Intensity | 3.74 | 1.37 |
| 9 | Younger | Musicians | Spectral env. | 3.16 | 2.14 |
| 9 | Younger | Musicians | Temporal env. | 1.72 | 2.06 |
| 9 | Younger | Nonmusicians | Intensity | 2.60 | 1.83 |
| 9 | Younger | Nonmusicians | Spectral env. | 1.89 | 2.06 |
| 9 | Younger | Nonmusicians | Temporal env. | 0.60 | 1.26 |
| 10 | Older | Musicians | Intensity | 3.81 | 1.68 |
| 10 | Older | Musicians | Spectral env. | 2.28 | 1.94 |
| 10 | Older | Musicians | Temporal env. | 1.16 | 1.34 |
| 10 | Older | Nonmusicians | Intensity | 3.38 | 1.94 |
| 10 | Older | Nonmusicians | Spectral env. | 1.64 | 2.25 |
| 10 | Older | Nonmusicians | Temporal env. | 0.61 | 1.43 |
| 10 | Younger | Musicians | Intensity | 3.71 | 1.70 |
| 10 | Younger | Musicians | Spectral env. | 3.20 | 2.15 |
| 10 | Younger | Musicians | Temporal env. | 1.76 | 2.11 |
| 10 | Younger | Nonmusicians | Intensity | 2.78 | 1.84 |
| 10 | Younger | Nonmusicians | Spectral env. | 1.44 | 2.14 |
| 10 | Younger | Nonmusicians | Temporal env. | 0.38 | 1.74 |
| 11 | Older | Musicians | Intensity | 3.55 | 1.53 |
| 11 | Older | Musicians | Spectral env. | 1.80 | 1.97 |
| 11 | Older | Musicians | Temporal env. | 1.05 | 1.59 |
| 11 | Older | Nonmusicians | Intensity | 3.05 | 1.89 |
| 11 | Older | Nonmusicians | Spectral env. | 0.83 | 1.99 |
| 11 | Older | Nonmusicians | Temporal env. | 0.47 | 1.20 |
| 11 | Younger | Musicians | Intensity | 3.41 | 1.56 |
| 11 | Younger | Musicians | Spectral env. | 2.76 | 2.10 |
| 11 | Younger | Musicians | Temporal env. | 1.78 | 1.94 |
| 11 | Younger | Nonmusicians | Intensity | 1.47 | 1.75 |
| 11 | Younger | Nonmusicians | Spectral env. | 1.66 | 1.88 |
| 11 | Younger | Nonmusicians | Temporal env. | 0.44 | 1.18 |
| 12 | Older | Musicians | Intensity | 3.32 | 2.02 |
| 12 | Older | Musicians | Spectral env. | 1.42 | 2.17 |
| 12 | Older | Musicians | Temporal env. | 1.03 | 1.50 |
| 12 | Older | Nonmusicians | Intensity | 3.26 | 1.64 |
| 12 | Older | Nonmusicians | Spectral env. | 0.81 | 1.82 |
| 12 | Older | Nonmusicians | Temporal env. | 0.94 | 1.70 |
| 12 | Younger | Musicians | Intensity | 3.50 | 1.74 |
| 12 | Younger | Musicians | Spectral env. | 2.54 | 2.29 |
| 12 | Younger | Musicians | Temporal env. | 1.73 | 2.07 |
| 12 | Younger | Nonmusicians | Intensity | 1.56 | 2.06 |
| 12 | Younger | Nonmusicians | Spectral env. | 1.54 | 2.36 |
| 12 | Younger | Nonmusicians | Temporal env. | 0.39 | 1.32 |
| 13 | Older | Musicians | Intensity | 3.22 | 1.80 |
| 13 | Older | Musicians | Spectral env. | 1.11 | 1.65 |
| 13 | Older | Musicians | Temporal env. | 0.90 | 1.84 |
| 13 | Older | Nonmusicians | Intensity | 2.82 | 2.05 |
| 13 | Older | Nonmusicians | Spectral env. | 1.14 | 1.83 |
| 13 | Older | Nonmusicians | Temporal env. | 0.99 | 1.33 |
| 13 | Younger | Musicians | Intensity | 3.42 | 2.06 |
| 13 | Younger | Musicians | Spectral env. | 2.16 | 2.30 |
| 13 | Younger | Musicians | Temporal env. | 1.49 | 1.63 |
| 13 | Younger | Nonmusicians | Intensity | 1.60 | 1.91 |
| 13 | Younger | Nonmusicians | Spectral env. | 1.56 | 1.91 |
| 13 | Younger | Nonmusicians | Temporal env. | 0.12 | 1.34 |
| 14 | Older | Musicians | Intensity | 3.12 | 1.88 |
| 14 | Older | Musicians | Spectral env. | 0.93 | 1.18 |
| 14 | Older | Musicians | Temporal env. | 0.32 | 1.42 |
| 14 | Older | Nonmusicians | Intensity | 2.37 | 2.33 |
| 14 | Older | Nonmusicians | Spectral env. | 0.82 | 1.47 |
| 14 | Older | Nonmusicians | Temporal env. | 0.73 | 1.47 |
| 14 | Younger | Musicians | Intensity | 3.12 | 2.13 |
| 14 | Younger | Musicians | Spectral env. | 1.83 | 2.20 |
| 14 | Younger | Musicians | Temporal env. | 1.72 | 1.70 |
| 14 | Younger | Nonmusicians | Intensity | 1.28 | 2.09 |
| 14 | Younger | Nonmusicians | Spectral env. | 1.29 | 1.69 |
| 14 | Younger | Nonmusicians | Temporal env. | 0.21 | 0.73 |
| 15 | Older | Musicians | Intensity | 2.25 | 2.11 |
| 15 | Older | Musicians | Spectral env. | 0.43 | 1.34 |
| 15 | Older | Musicians | Temporal env. | 0.90 | 1.36 |
| 15 | Older | Nonmusicians | Intensity | 2.10 | 2.17 |
| 15 | Older | Nonmusicians | Spectral env. | 0.51 | 1.60 |
| 15 | Older | Nonmusicians | Temporal env. | 0.26 | 0.66 |
| 15 | Younger | Musicians | Intensity | 2.99 | 1.85 |
| 15 | Younger | Musicians | Spectral env. | 1.57 | 2.27 |
| 15 | Younger | Musicians | Temporal env. | 1.61 | 1.83 |
| 15 | Younger | Nonmusicians | Intensity | 1.23 | 1.88 |
| 15 | Younger | Nonmusicians | Spectral env. | 1.03 | 1.64 |
| 15 | Younger | Nonmusicians | Temporal env. | 0.74 | 1.21 |
| 16 | Older | Musicians | Intensity | 1.82 | 1.99 |
| 16 | Older | Musicians | Spectral env. | 0.92 | 1.78 |
| 16 | Older | Musicians | Temporal env. | 0.99 | 1.49 |
| 16 | Older | Nonmusicians | Intensity | 2.05 | 1.77 |
| 16 | Older | Nonmusicians | Spectral env. | 0.05 | 0.74 |
| 16 | Older | Nonmusicians | Temporal env. | 0.54 | 1.22 |
| 16 | Younger | Musicians | Intensity | 2.99 | 2.06 |
| 16 | Younger | Musicians | Spectral env. | 1.36 | 1.98 |
| 16 | Younger | Musicians | Temporal env. | 1.75 | 1.94 |
| 16 | Younger | Nonmusicians | Intensity | 1.07 | 1.67 |
| 16 | Younger | Nonmusicians | Spectral env. | 0.74 | 1.76 |
| 16 | Younger | Nonmusicians | Temporal env. | 0.63 | 1.11 |
| 17 | Older | Musicians | Intensity | 1.54 | 1.86 |
| 17 | Older | Musicians | Spectral env. | 0.82 | 1.42 |
| 17 | Older | Musicians | Temporal env. | 0.23 | 1.13 |
| 17 | Older | Nonmusicians | Intensity | 1.30 | 1.47 |
| 17 | Older | Nonmusicians | Spectral env. | 0.25 | 1.19 |
| 17 | Older | Nonmusicians | Temporal env. | 0.52 | 0.78 |
| 17 | Younger | Musicians | Intensity | 2.54 | 2.33 |
| 17 | Younger | Musicians | Spectral env. | 1.37 | 1.59 |
| 17 | Younger | Musicians | Temporal env. | 1.84 | 1.86 |
| 17 | Younger | Nonmusicians | Intensity | 1.02 | 1.90 |
| 17 | Younger | Nonmusicians | Spectral env. | 0.13 | 1.41 |
| 17 | Younger | Nonmusicians | Temporal env. | 0.31 | 1.15 |
| 18 | Older | Musicians | Intensity | 1.09 | 1.45 |
| 18 | Older | Musicians | Spectral env. | 0.85 | 1.18 |
| 18 | Older | Musicians | Temporal env. | 0.56 | 0.83 |
| 18 | Older | Nonmusicians | Intensity | 0.51 | 1.08 |
| 18 | Older | Nonmusicians | Spectral env. | 0.50 | 1.15 |
| 18 | Older | Nonmusicians | Temporal env. | 0.65 | 1.37 |
| 18 | Younger | Musicians | Intensity | 2.55 | 1.93 |
| 18 | Younger | Musicians | Spectral env. | 1.44 | 2.00 |
| 18 | Younger | Musicians | Temporal env. | 1.61 | 1.96 |
| 18 | Younger | Nonmusicians | Intensity | 0.75 | 1.42 |
| 18 | Younger | Nonmusicians | Spectral env. | 0.34 | 0.95 |
| 18 | Younger | Nonmusicians | Temporal env. | 0.23 | 0.85 |
| 19 | Older | Musicians | Intensity | 1.09 | 1.86 |
| 19 | Older | Musicians | Spectral env. | 0.64 | 1.37 |
| 19 | Older | Musicians | Temporal env. | 0.33 | 0.92 |
| 19 | Older | Nonmusicians | Intensity | 0.49 | 0.88 |
| 19 | Older | Nonmusicians | Spectral env. | 0.86 | 1.49 |
| 19 | Older | Nonmusicians | Temporal env. | 0.36 | 1.09 |
| 19 | Younger | Musicians | Intensity | 1.88 | 2.06 |
| 19 | Younger | Musicians | Spectral env. | 1.36 | 2.11 |
| 19 | Younger | Musicians | Temporal env. | 1.34 | 1.84 |
| 19 | Younger | Nonmusicians | Intensity | 0.59 | 1.30 |
| 19 | Younger | Nonmusicians | Spectral env. | 0.37 | 1.22 |
| 19 | Younger | Nonmusicians | Temporal env. | 0.34 | 1.07 |
| 20 | Older | Musicians | Intensity | 1.53 | 1.71 |
| 20 | Older | Musicians | Spectral env. | 0.68 | 1.34 |
| 20 | Older | Musicians | Temporal env. | 0.26 | 0.91 |
| 20 | Older | Nonmusicians | Intensity | 0.01 | 0.60 |
| 20 | Older | Nonmusicians | Spectral env. | 0.14 | 0.96 |
| 20 | Older | Nonmusicians | Temporal env. | 0.16 | 0.96 |
| 20 | Younger | Musicians | Intensity | 1.65 | 1.74 |
| 20 | Younger | Musicians | Spectral env. | 1.42 | 1.72 |
| 20 | Younger | Musicians | Temporal env. | 1.35 | 1.96 |
| 20 | Younger | Nonmusicians | Intensity | 0.65 | 1.57 |
| 20 | Younger | Nonmusicians | Spectral env. | 0.09 | 0.94 |
| 20 | Younger | Nonmusicians | Temporal env. | 0.31 | 1.78 |

#### Table 2. Summary statistics (mean, standard deviation) of transformed d’ by level, age group, musicianship, and feature.

| Level | Age Group | Musicianship | Feature | Mean transformed d’ | SD transformed d’ |
| --- | --- | --- | --- | --- | --- |
| 0.00 | Younger | Nonmusician | Intensity | 0.65 | 1.57 |
| 0.00 | Younger | Nonmusician | Spectral Envelope | 0.09 | 0.94 |
| 0.00 | Younger | Nonmusician | Temporal Envelope | 0.31 | 1.78 |
| 0.00 | Younger | Musician | Intensity | 1.65 | 1.74 |
| 0.00 | Younger | Musician | Spectral Envelope | 1.42 | 1.72 |
| 0.00 | Younger | Musician | Temporal Envelope | 1.35 | 1.96 |
| 0.00 | Older | Nonmusician | Intensity | 0.01 | 0.60 |
| 0.00 | Older | Nonmusician | Spectral Envelope | 0.14 | 0.96 |
| 0.00 | Older | Nonmusician | Temporal Envelope | 0.16 | 0.96 |
| 0.00 | Older | Musician | Intensity | 1.53 | 1.71 |
| 0.00 | Older | Musician | Spectral Envelope | 0.68 | 1.34 |
| 0.00 | Older | Musician | Temporal Envelope | 0.26 | 0.91 |
| 0.06 | Younger | Nonmusician | Temporal Envelope | 0.34 | 1.07 |
| 0.06 | Younger | Musician | Temporal Envelope | 1.34 | 1.84 |
| 0.06 | Older | Nonmusician | Temporal Envelope | 0.36 | 1.09 |
| 0.06 | Older | Musician | Temporal Envelope | 0.33 | 0.92 |
| 0.12 | Younger | Nonmusician | Intensity | 0.59 | 1.30 |
| 0.12 | Younger | Musician | Intensity | 1.88 | 2.06 |
| 0.12 | Older | Nonmusician | Intensity | 0.49 | 0.88 |
| 0.12 | Older | Musician | Intensity | 1.09 | 1.86 |
| 0.12 | Younger | Nonmusician | Temporal Envelope | 0.23 | 0.85 |
| 0.12 | Younger | Musician | Temporal Envelope | 1.61 | 1.96 |
| 0.12 | Older | Nonmusician | Temporal Envelope | 0.65 | 1.37 |
| 0.12 | Older | Musician | Temporal Envelope | 0.56 | 0.83 |
| 0.13 | Younger | Nonmusician | Spectral Envelope | 0.37 | 1.22 |
| 0.13 | Younger | Musician | Spectral Envelope | 1.36 | 2.11 |
| 0.13 | Older | Nonmusician | Spectral Envelope | 0.86 | 1.49 |
| 0.13 | Older | Musician | Spectral Envelope | 0.64 | 1.37 |
| 0.18 | Younger | Nonmusician | Temporal Envelope | 0.31 | 1.15 |
| 0.18 | Younger | Musician | Temporal Envelope | 1.84 | 1.86 |
| 0.18 | Older | Nonmusician | Temporal Envelope | 0.52 | 0.78 |
| 0.18 | Older | Musician | Temporal Envelope | 0.23 | 1.13 |
| 0.24 | Younger | Nonmusician | Intensity | 0.75 | 1.42 |
| 0.24 | Younger | Musician | Intensity | 2.55 | 1.93 |
| 0.24 | Older | Nonmusician | Intensity | 0.51 | 1.08 |
| 0.24 | Older | Musician | Intensity | 1.09 | 1.45 |
| 0.24 | Younger | Nonmusician | Temporal Envelope | 0.63 | 1.11 |
| 0.24 | Younger | Musician | Temporal Envelope | 1.75 | 1.94 |
| 0.24 | Older | Nonmusician | Temporal Envelope | 0.54 | 1.22 |
| 0.24 | Older | Musician | Temporal Envelope | 0.99 | 1.49 |
| 0.26 | Younger | Nonmusician | Spectral Envelope | 0.34 | 0.95 |
| 0.26 | Younger | Musician | Spectral Envelope | 1.44 | 2.00 |
| 0.26 | Older | Nonmusician | Spectral Envelope | 0.50 | 1.15 |
| 0.26 | Older | Musician | Spectral Envelope | 0.85 | 1.18 |
| 0.30 | Younger | Nonmusician | Temporal Envelope | 0.74 | 1.21 |
| 0.30 | Younger | Musician | Temporal Envelope | 1.61 | 1.83 |
| 0.30 | Older | Nonmusician | Temporal Envelope | 0.26 | 0.66 |
| 0.30 | Older | Musician | Temporal Envelope | 0.90 | 1.36 |
| 0.36 | Younger | Nonmusician | Temporal Envelope | 0.21 | 0.73 |
| 0.36 | Younger | Musician | Temporal Envelope | 1.72 | 1.70 |
| 0.36 | Older | Nonmusician | Temporal Envelope | 0.73 | 1.47 |
| 0.36 | Older | Musician | Temporal Envelope | 0.32 | 1.42 |
| 0.36 | Younger | Nonmusician | Intensity | 1.02 | 1.90 |
| 0.36 | Younger | Musician | Intensity | 2.54 | 2.33 |
| 0.36 | Older | Nonmusician | Intensity | 1.30 | 1.47 |
| 0.36 | Older | Musician | Intensity | 1.54 | 1.86 |
| 0.40 | Younger | Nonmusician | Spectral Envelope | 0.13 | 1.41 |
| 0.40 | Younger | Musician | Spectral Envelope | 1.37 | 1.59 |
| 0.40 | Older | Nonmusician | Spectral Envelope | 0.25 | 1.19 |
| 0.40 | Older | Musician | Spectral Envelope | 0.82 | 1.42 |
| 0.42 | Younger | Nonmusician | Temporal Envelope | 0.12 | 1.34 |
| 0.42 | Younger | Musician | Temporal Envelope | 1.49 | 1.63 |
| 0.42 | Older | Nonmusician | Temporal Envelope | 0.99 | 1.33 |
| 0.42 | Older | Musician | Temporal Envelope | 0.90 | 1.84 |
| 0.47 | Younger | Nonmusician | Temporal Envelope | 0.39 | 1.32 |
| 0.47 | Younger | Musician | Temporal Envelope | 1.73 | 2.07 |
| 0.47 | Older | Nonmusician | Temporal Envelope | 0.94 | 1.70 |
| 0.47 | Older | Musician | Temporal Envelope | 1.03 | 1.50 |
| 0.48 | Younger | Nonmusician | Intensity | 1.07 | 1.67 |
| 0.48 | Younger | Musician | Intensity | 2.99 | 2.06 |
| 0.48 | Older | Nonmusician | Intensity | 2.05 | 1.77 |
| 0.48 | Older | Musician | Intensity | 1.82 | 1.99 |
| 0.53 | Younger | Nonmusician | Temporal Envelope | 0.44 | 1.18 |
| 0.53 | Younger | Musician | Temporal Envelope | 1.78 | 1.94 |
| 0.53 | Older | Nonmusician | Temporal Envelope | 0.47 | 1.20 |
| 0.53 | Older | Musician | Temporal Envelope | 1.05 | 1.59 |
| 0.55 | Younger | Nonmusician | Spectral Envelope | 0.74 | 1.76 |
| 0.55 | Younger | Musician | Spectral Envelope | 1.36 | 1.98 |
| 0.55 | Older | Nonmusician | Spectral Envelope | 0.05 | 0.74 |
| 0.55 | Older | Musician | Spectral Envelope | 0.92 | 1.78 |
| 0.58 | Younger | Nonmusician | Temporal Envelope | 0.38 | 1.74 |
| 0.58 | Younger | Musician | Temporal Envelope | 1.76 | 2.11 |
| 0.58 | Older | Nonmusician | Temporal Envelope | 0.61 | 1.43 |
| 0.58 | Older | Musician | Temporal Envelope | 1.16 | 1.34 |
| 0.60 | Younger | Nonmusician | Intensity | 1.23 | 1.88 |
| 0.60 | Younger | Musician | Intensity | 2.99 | 1.85 |
| 0.60 | Older | Nonmusician | Intensity | 2.10 | 2.17 |
| 0.60 | Older | Musician | Intensity | 2.25 | 2.11 |
| 0.63 | Younger | Nonmusician | Temporal Envelope | 0.60 | 1.26 |
| 0.63 | Younger | Musician | Temporal Envelope | 1.72 | 2.06 |
| 0.63 | Older | Nonmusician | Temporal Envelope | 0.44 | 1.32 |
| 0.63 | Older | Musician | Temporal Envelope | 0.89 | 1.88 |
| 0.69 | Younger | Nonmusician | Temporal Envelope | 0.72 | 1.79 |
| 0.69 | Younger | Musician | Temporal Envelope | 1.47 | 2.10 |
| 0.69 | Older | Nonmusician | Temporal Envelope | 0.67 | 1.20 |
| 0.69 | Older | Musician | Temporal Envelope | 1.74 | 1.87 |
| 0.70 | Younger | Nonmusician | Spectral Envelope | 1.03 | 1.64 |
| 0.70 | Younger | Musician | Spectral Envelope | 1.57 | 2.27 |
| 0.70 | Older | Nonmusician | Spectral Envelope | 0.51 | 1.60 |
| 0.70 | Older | Musician | Spectral Envelope | 0.43 | 1.34 |
| 0.72 | Younger | Nonmusician | Intensity | 1.28 | 2.09 |
| 0.72 | Younger | Musician | Intensity | 3.12 | 2.13 |
| 0.72 | Older | Nonmusician | Intensity | 2.37 | 2.33 |
| 0.72 | Older | Musician | Intensity | 3.12 | 1.88 |
| 0.74 | Younger | Nonmusician | Temporal Envelope | 0.70 | 1.59 |
| 0.74 | Younger | Musician | Temporal Envelope | 1.88 | 2.12 |
| 0.74 | Older | Nonmusician | Temporal Envelope | 0.52 | 1.43 |
| 0.74 | Older | Musician | Temporal Envelope | 1.06 | 2.09 |
| 0.79 | Younger | Nonmusician | Temporal Envelope | 0.67 | 1.89 |
| 0.79 | Younger | Musician | Temporal Envelope | 1.95 | 2.22 |
| 0.79 | Older | Nonmusician | Temporal Envelope | 1.04 | 1.56 |
| 0.79 | Older | Musician | Temporal Envelope | 1.25 | 1.49 |
| 0.84 | Younger | Nonmusician | Temporal Envelope | 0.37 | 1.41 |
| 0.84 | Younger | Musician | Temporal Envelope | 2.03 | 1.95 |
| 0.84 | Older | Nonmusician | Temporal Envelope | 0.98 | 1.96 |
| 0.84 | Older | Musician | Temporal Envelope | 1.13 | 1.76 |
| 0.84 | Younger | Nonmusician | Intensity | 1.60 | 1.91 |
| 0.84 | Younger | Musician | Intensity | 3.42 | 2.06 |
| 0.84 | Older | Nonmusician | Intensity | 2.82 | 2.05 |
| 0.84 | Older | Musician | Intensity | 3.22 | 1.80 |
| 0.86 | Younger | Nonmusician | Spectral Envelope | 1.29 | 1.69 |
| 0.86 | Younger | Musician | Spectral Envelope | 1.83 | 2.20 |
| 0.86 | Older | Nonmusician | Spectral Envelope | 0.82 | 1.47 |
| 0.86 | Older | Musician | Spectral Envelope | 0.93 | 1.18 |
| 0.88 | Younger | Nonmusician | Temporal Envelope | 0.54 | 1.22 |
| 0.88 | Younger | Musician | Temporal Envelope | 2.47 | 2.21 |
| 0.88 | Older | Nonmusician | Temporal Envelope | 0.80 | 1.76 |
| 0.88 | Older | Musician | Temporal Envelope | 1.48 | 1.85 |
| 0.93 | Younger | Nonmusician | Temporal Envelope | 0.76 | 1.49 |
| 0.93 | Younger | Musician | Temporal Envelope | 2.78 | 2.08 |
| 0.93 | Older | Nonmusician | Temporal Envelope | 0.94 | 1.69 |
| 0.93 | Older | Musician | Temporal Envelope | 1.15 | 1.89 |
| 0.96 | Younger | Nonmusician | Intensity | 1.56 | 2.06 |
| 0.96 | Younger | Musician | Intensity | 3.50 | 1.74 |
| 0.96 | Older | Nonmusician | Intensity | 3.26 | 1.64 |
| 0.96 | Older | Musician | Intensity | 3.32 | 2.02 |
| 0.97 | Younger | Nonmusician | Temporal Envelope | 0.68 | 1.61 |
| 0.97 | Younger | Musician | Temporal Envelope | 2.38 | 2.21 |
| 0.97 | Older | Nonmusician | Temporal Envelope | 1.15 | 1.41 |
| 0.97 | Older | Musician | Temporal Envelope | 0.83 | 2.05 |
| 1.02 | Younger | Nonmusician | Temporal Envelope | 0.74 | 1.70 |
| 1.02 | Younger | Musician | Temporal Envelope | 2.16 | 2.32 |
| 1.02 | Older | Nonmusician | Temporal Envelope | 1.11 | 1.79 |
| 1.02 | Older | Musician | Temporal Envelope | 1.75 | 1.77 |
| 1.03 | Younger | Nonmusician | Spectral Envelope | 1.56 | 1.91 |
| 1.03 | Younger | Musician | Spectral Envelope | 2.16 | 2.30 |
| 1.03 | Older | Nonmusician | Spectral Envelope | 1.14 | 1.83 |
| 1.03 | Older | Musician | Spectral Envelope | 1.11 | 1.65 |
| 1.08 | Younger | Nonmusician | Intensity | 1.47 | 1.75 |
| 1.08 | Younger | Musician | Intensity | 3.41 | 1.56 |
| 1.08 | Older | Nonmusician | Intensity | 3.05 | 1.89 |
| 1.08 | Older | Musician | Intensity | 3.55 | 1.53 |
| 1.20 | Younger | Nonmusician | Intensity | 2.78 | 1.84 |
| 1.20 | Younger | Musician | Intensity | 3.71 | 1.70 |
| 1.20 | Older | Nonmusician | Intensity | 3.38 | 1.94 |
| 1.20 | Older | Musician | Intensity | 3.81 | 1.68 |
| 1.21 | Younger | Nonmusician | Spectral Envelope | 1.54 | 2.36 |
| 1.21 | Younger | Musician | Spectral Envelope | 2.54 | 2.29 |
| 1.21 | Older | Nonmusician | Spectral Envelope | 0.81 | 1.82 |
| 1.21 | Older | Musician | Spectral Envelope | 1.42 | 2.17 |
| 1.32 | Younger | Nonmusician | Intensity | 2.60 | 1.83 |
| 1.32 | Younger | Musician | Intensity | 3.74 | 1.37 |
| 1.32 | Older | Nonmusician | Intensity | 3.41 | 1.61 |
| 1.32 | Older | Musician | Intensity | 3.73 | 1.57 |
| 1.40 | Younger | Nonmusician | Spectral Envelope | 1.66 | 1.88 |
| 1.40 | Younger | Musician | Spectral Envelope | 2.76 | 2.10 |
| 1.40 | Older | Nonmusician | Spectral Envelope | 0.83 | 1.99 |
| 1.40 | Older | Musician | Spectral Envelope | 1.80 | 1.97 |
| 1.44 | Younger | Nonmusician | Intensity | 2.52 | 1.79 |
| 1.44 | Younger | Musician | Intensity | 3.51 | 1.55 |
| 1.44 | Older | Nonmusician | Intensity | 3.65 | 1.86 |
| 1.44 | Older | Musician | Intensity | 3.86 | 1.49 |
| 1.56 | Younger | Nonmusician | Intensity | 2.58 | 2.14 |
| 1.56 | Younger | Musician | Intensity | 3.76 | 1.41 |
| 1.56 | Older | Nonmusician | Intensity | 3.70 | 1.79 |
| 1.56 | Older | Musician | Intensity | 4.09 | 1.19 |
| 1.60 | Younger | Nonmusician | Spectral Envelope | 1.44 | 2.14 |
| 1.60 | Younger | Musician | Spectral Envelope | 3.20 | 2.15 |
| 1.60 | Older | Nonmusician | Spectral Envelope | 1.64 | 2.25 |
| 1.60 | Older | Musician | Spectral Envelope | 2.28 | 1.94 |
| 1.68 | Younger | Nonmusician | Intensity | 2.49 | 1.83 |
| 1.68 | Younger | Musician | Intensity | 3.70 | 1.67 |
| 1.68 | Older | Nonmusician | Intensity | 3.33 | 1.83 |
| 1.68 | Older | Musician | Intensity | 4.03 | 1.37 |
| 1.80 | Younger | Nonmusician | Intensity | 2.69 | 1.83 |
| 1.80 | Younger | Musician | Intensity | 3.74 | 1.43 |
| 1.80 | Older | Nonmusician | Intensity | 3.60 | 1.52 |
| 1.80 | Older | Musician | Intensity | 4.17 | 1.13 |
| 1.80 | Younger | Nonmusician | Spectral Envelope | 1.89 | 2.06 |
| 1.80 | Younger | Musician | Spectral Envelope | 3.16 | 2.14 |
| 1.80 | Older | Nonmusician | Spectral Envelope | 1.62 | 2.07 |
| 1.80 | Older | Musician | Spectral Envelope | 2.00 | 2.10 |
| 1.92 | Younger | Nonmusician | Intensity | 2.80 | 1.87 |
| 1.92 | Younger | Musician | Intensity | 3.97 | 1.32 |
| 1.92 | Older | Nonmusician | Intensity | 3.46 | 1.52 |
| 1.92 | Older | Musician | Intensity | 4.05 | 1.22 |
| 2.02 | Younger | Nonmusician | Spectral Envelope | 1.84 | 1.96 |
| 2.02 | Younger | Musician | Spectral Envelope | 3.06 | 1.97 |
| 2.02 | Older | Nonmusician | Spectral Envelope | 2.18 | 1.97 |
| 2.02 | Older | Musician | Spectral Envelope | 2.09 | 2.10 |
| 2.04 | Younger | Nonmusician | Intensity | 2.76 | 1.82 |
| 2.04 | Younger | Musician | Intensity | 4.19 | 0.96 |
| 2.04 | Older | Nonmusician | Intensity | 3.66 | 1.43 |
| 2.04 | Older | Musician | Intensity | 4.08 | 1.22 |
| 2.16 | Younger | Nonmusician | Intensity | 2.76 | 1.71 |
| 2.16 | Younger | Musician | Intensity | 3.96 | 1.02 |
| 2.16 | Older | Nonmusician | Intensity | 3.41 | 1.64 |
| 2.16 | Older | Musician | Intensity | 3.98 | 1.28 |
| 2.25 | Younger | Nonmusician | Spectral Envelope | 1.84 | 2.08 |
| 2.25 | Younger | Musician | Spectral Envelope | 3.03 | 2.06 |
| 2.25 | Older | Nonmusician | Spectral Envelope | 2.44 | 2.19 |
| 2.25 | Older | Musician | Spectral Envelope | 2.27 | 2.02 |
| 2.28 | Younger | Nonmusician | Intensity | 2.88 | 1.90 |
| 2.28 | Younger | Musician | Intensity | 3.98 | 1.61 |
| 2.28 | Older | Nonmusician | Intensity | 3.35 | 1.85 |
| 2.28 | Older | Musician | Intensity | 4.04 | 1.39 |
| 2.49 | Younger | Nonmusician | Spectral Envelope | 2.38 | 1.99 |
| 2.49 | Younger | Musician | Spectral Envelope | 3.54 | 1.66 |
| 2.49 | Older | Nonmusician | Spectral Envelope | 2.22 | 2.16 |
| 2.49 | Older | Musician | Spectral Envelope | 2.52 | 2.25 |
| 2.74 | Younger | Nonmusician | Spectral Envelope | 1.97 | 2.09 |
| 2.74 | Younger | Musician | Spectral Envelope | 3.32 | 2.08 |
| 2.74 | Older | Nonmusician | Spectral Envelope | 2.27 | 2.40 |
| 2.74 | Older | Musician | Spectral Envelope | 2.58 | 2.05 |
| 3.00 | Younger | Nonmusician | Spectral Envelope | 1.83 | 1.82 |
| 3.00 | Younger | Musician | Spectral Envelope | 3.55 | 1.68 |
| 3.00 | Older | Nonmusician | Spectral Envelope | 2.43 | 2.08 |
| 3.00 | Older | Musician | Spectral Envelope | 2.55 | 2.33 |
| 3.28 | Younger | Nonmusician | Spectral Envelope | 1.79 | 2.31 |
| 3.28 | Younger | Musician | Spectral Envelope | 3.67 | 1.61 |
| 3.28 | Older | Nonmusician | Spectral Envelope | 2.54 | 2.02 |
| 3.28 | Older | Musician | Spectral Envelope | 2.82 | 2.37 |
| 3.57 | Younger | Nonmusician | Spectral Envelope | 2.22 | 2.30 |
| 3.57 | Younger | Musician | Spectral Envelope | 3.79 | 1.64 |
| 3.57 | Older | Nonmusician | Spectral Envelope | 2.71 | 1.94 |
| 3.57 | Older | Musician | Spectral Envelope | 2.59 | 2.19 |
| 3.87 | Younger | Nonmusician | Spectral Envelope | 1.84 | 1.93 |
| 3.87 | Younger | Musician | Spectral Envelope | 3.48 | 1.64 |
| 3.87 | Older | Nonmusician | Spectral Envelope | 2.52 | 2.10 |
| 3.87 | Older | Musician | Spectral Envelope | 3.01 | 1.88 |
